# Cryogenic Infrared Spectroscopy Unmasks Gas‐Phase Charge Migration in Mucin‐Type *O*‐Glycans

**DOI:** 10.1002/smll.202600077

**Published:** 2026-05-15

**Authors:** Marc Safferthal, Gurpur Rakesh D. Prabhu, América Y. Torres‐Boy, Jerome Riedel, Leïla Bechtella, Wesley Pietsch, Gerard Meijer, Gert von Helden, Gaël M. Vos, Kevin Pagel

**Affiliations:** ^1^ Department of Biology, Chemistry, Pharmacy Freie Universität Berlin Berlin Germany; ^2^ Department of Molecular Physics Fritz Haber Institute of the Max Planck Society Berlin Germany

**Keywords:** charge migration, density functional theory, ion mobility spectrometry, IR spectroscopy, O‐glycans

## Abstract

*O*‐glycosylation is an essential post‐translational modification, controlling biological processes like innate immunity, lubrication, and cell communication. Due to the inherent structural complexity of *O*‐glycans, assigning exact structures by mass spectrometry is highly challenging. Detailed structural assignment involves laborious processing and data interpretation but often remains incomplete due to the absence of diagnostic fragments. In this work, we introduce cryogenic gas‐phase infrared spectroscopy to facilitate the characterization of *O*‐glycans. We show *O*‐glycans provide highly diagnostic IR fingerprints, which allow us to easily distinguish between isomeric *O*‐glycan core structures. *Ab initio* calculations are used to understand the gas‐phase structures of the deprotonated *O*‐glycans. The experimental spectra suggest contributions from distinct ensembles of deprotomers and conformers present at cryogenic temperatures in the gas phase that are not detected in ion mobility spectrometry experiments at ambient temperature. We find that rapid charge migration processes, reminiscent of the Grotthuss mechanism in water, allows interconversion of deprotomers at ambient temperatures due to the presence of dense hydrogen‐bond networks and the high flexibility of *O*‐glycans in the gas phase.

## Graphical abstract

Protons in a sugar rush: *O*‐glycans are vital biomolecules, challenging to analyze due to isomeric forms. Using ion mobility spectrometry, cryogenic IR spectroscopy, and *ab initio* calculations, isomeric *O*‐glycan cores can be distinguished. At cryogenic temperatures, multiple deprotomers were identified, which interconvert at ambient conditions via rapid gas‐phase charge migration along internal hydrogen bonds, reminiscent of the Grotthuss mechanism.

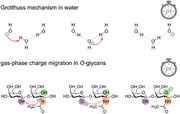

## 1 Introduction

**FIGURE 1:**
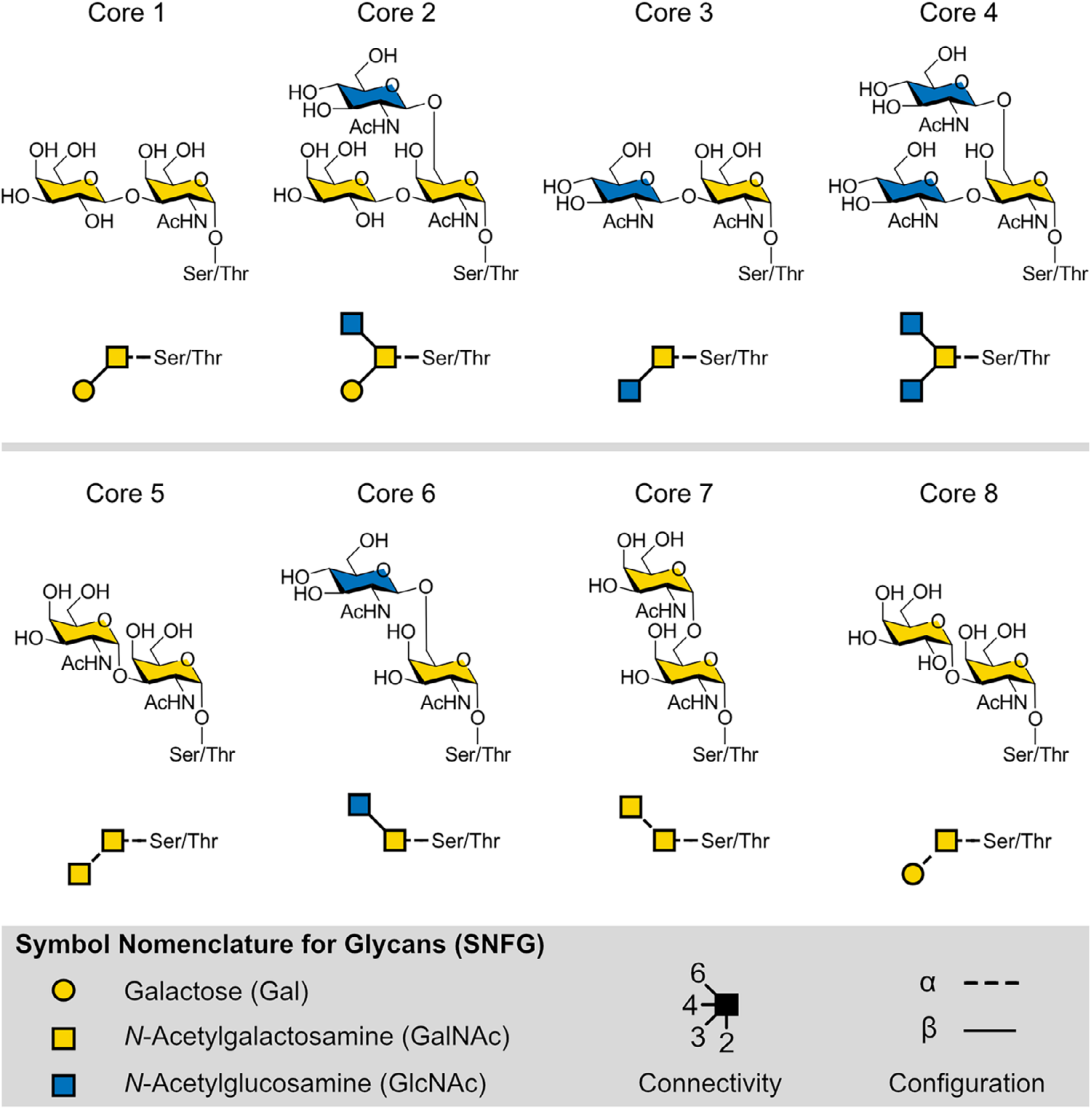
Mucin‐Type *O*‐glycan cores depicted as chemical structures and as representations following the Symbol Nomenclature for Glycans (SNFG).[5] Core 1–4 are the most common structures and core 5–8 are less abundant. Cores 1 and 8, as well as, cores 3,5,6, and 7 are isomers that differ in their glycosidic linkage. *O*‐glycan cores are commonly extended by *N*‐acetyllactosamine, sialic acid and fucose units to form more complex structures.

*O*‐glycosylation is a structurally diverse post‐translational modification found in most living organisms. Glucose, galactose, mannose, fucose, xylose, *N*‐acetylglucosamine or *N*‐acetylgalactosamine can be directly attached to proteins through the oxygen atom of an amino acid sidechain (mostly serine or threonine). *O*‐glycans that are attached through an *N*‐acetylgalactosamine unit, often called mucin‐type *O*‐glycans, represent the most common variant of *O*‐glycosylation [1]. Found on many glycopeptides and proteins such as mucins, peptide hormones, and blood proteins, they are involved in key biological processes like lubrication, innate immunity, receptor interactions, and protein homeostasis [2, 3, 4]. In contrast to *N*‐glycans that all share a single core structure, mucin‐type *O*‐glycans have eight different core structures, which greatly increases the structural complexity (Figure 1) [5]. Cores 1–4 are common in mammals and expressed by almost all cells whereas core 5–8 are less frequently observed [1]. The isomeric cores 3, 5, 6, and 7 differ by the composition of the second monosaccharide building block and the configuration of the glycosidic bond (*α*/*β* configurations). Core 1 and 8 represent isomers differing only in the orientation of the glycosidic bond. For a long time, these subtle differences could only be distinguished by a combination of preparative scale liquid chromatography (LC) and NMR spectroscopy [6, 7]. However, data acquisition and analysis of *O*‐glycans by NMR spectroscopy requires high amounts of purified material and significant expertise. The structural assignment of *O*‐glycosylation is especially challenging when dealing with biological samples, where only limited amounts of samples with highly heterogeneous glycans are common. The current go‐to methods rely on the separation of glycans using hydrophilic interaction liquid chromatography or porous graphitized carbon stationary phases [8, 9]. To characterize the separated *O*‐glycans, fragmentation is usually conducted at the MS^2^ level and spectra are matched with reference fragmentation spectra stored in databases [10]. The structural complexity of *O*‐glycans renders the detailed analysis very difficult despite the robustness of tandem MS approaches. Assignment requires expert knowledge on glycan structures and fragmentation behavior, although recent work has started to address the automation of this process using machine learning algorithms [11, 12]. The characterization of more complex structures remains prone to misassignment due to the absence of diagnostic fragments or the presence of repeated structural motifs that are difficult to distinguish. A key challenge in this assignment process is the confident characterization of the *O*‐glycan core structure.

To assign exact glycan structures including all linkage information, an additional dimension of information is often required. In order to generate this, ion mobility spectrometry (IMS) has been used to distinguish isomeric *O*‐glycans [13]. Formally corresponding to a gas‐phase electrophoresis, IMS separates glycans based on their size, shape, mass and charge. Especially, trapped ion mobility spectrometry (TIMS) achieves rapid isomer separation of *O*‐glycans in the gas phase on a millisecond timescale [9]. IMS can be combined with (LC‐)MS/MS and allows to measure rotationally‐averaged collision cross sections (CCS) of the ions, which correspond to the effective area involved in the interaction between ions and neutral gas molecules. This intrinsic property can vary for different isomeric structures and can be used for *O*‐glycan assignment if the CCS is unique for a certain isomer. However, not all isomers are readily distinguishable by CCS, especially since the number of potential isomers rapidly increases with structural complexity.

In this work, we introduce cryogenic gas‐phase infrared (IR) spectroscopy to the field of *O*‐glycan analysis. Over the past years, cryogenic gas‐phase IR spectroscopy emerged as a powerful tool to analyze glycan isomers like blood group epitopes [14, 15, 16, 17], human milk oligosaccharides [18, 19, 20, 21] and *N*‐glycans [22, 23]. Gas‐phase IR spectroscopy is highly sensitive to the spatial orientation of functional groups in a molecule making it a powerful tool to distinguish isomers. Especially, the highly diagnostic IR spectra obtained at cryogenic conditions allow for a rapid and simple glycan assignment by a database approach [22]. However, the cryogenic IR spectra of the *O*‐glycans we analyzed proved to be difficult to interpret. We find that no single structure explains the complex IR signatures obtained for the investigated glycans. Instead, the spectra are composed of multiple deprotomers that have unique spatial orientations dependent on rearranging internal hydrogen networks. By comparing experimental results obtained with cryogenic gas‐phase IR spectroscopy and IMS with *ab initio* calculations, we find that these networks can rapidly interconvert at ambient temperature due to charge migration.

## 2 Results and Discussion

### 2.1 Cryogenic Gas‐Phase IR Spectroscopy and TIMS of Isomeric *O*‐Glycans Cores

A critical step in *O*‐glycan analysis is the identification of the core architecture. However, *O*‐glycan cores 1/8 and 3/5/6/7 are isomers, which remain challenging to distinguish based on intrinsic information at the MS^1^ and MS^2^ level, especially in the absence of standards. To address this problem, we aimed to identify unique features of the core structures that would enable reliable and unambiguous characterization. Because of poor availability of the rare core 6 and 7 *O*‐glycans, we focus on the more common isomeric *O*‐glycan cores 1/8 and 3/5. In almost all *O*‐glycan analysis workflows, the glycan structure is released from the protein backbone. Since no enzymes are available that can efficiently release *O*‐glycans (such as PNGaseF for *N*‐glycans), chemical release methods are usually employed. Most common is a base‐mediated β‐elimination to liberate the *O*‐glycans as free reducing end glycans, altough oxidative strategies have also been reported recently [9, 24, 25, 26]. A common problem of β‐elimination release of *O*‐glycans is the so‐called ‘peeling reaction’, which leads to decomposition of *O*‐glycans. To prevent peeling, it is a common procedure to reduce the free reducing end glycan to glycan alditols [27]. An aditional benefit from this process is that reduction to alditols simplifies chromatographic separation, preventing peak‐broadening by the interconversion of *α*‐ and *β*‐anomers [9]. In‐line with the most common applied release strategy, we reduced free reducing end glycans containing *O*‐glycan cores 1, 3, 5, and 8 to the corresponding alditols.

**FIGURE 2:**
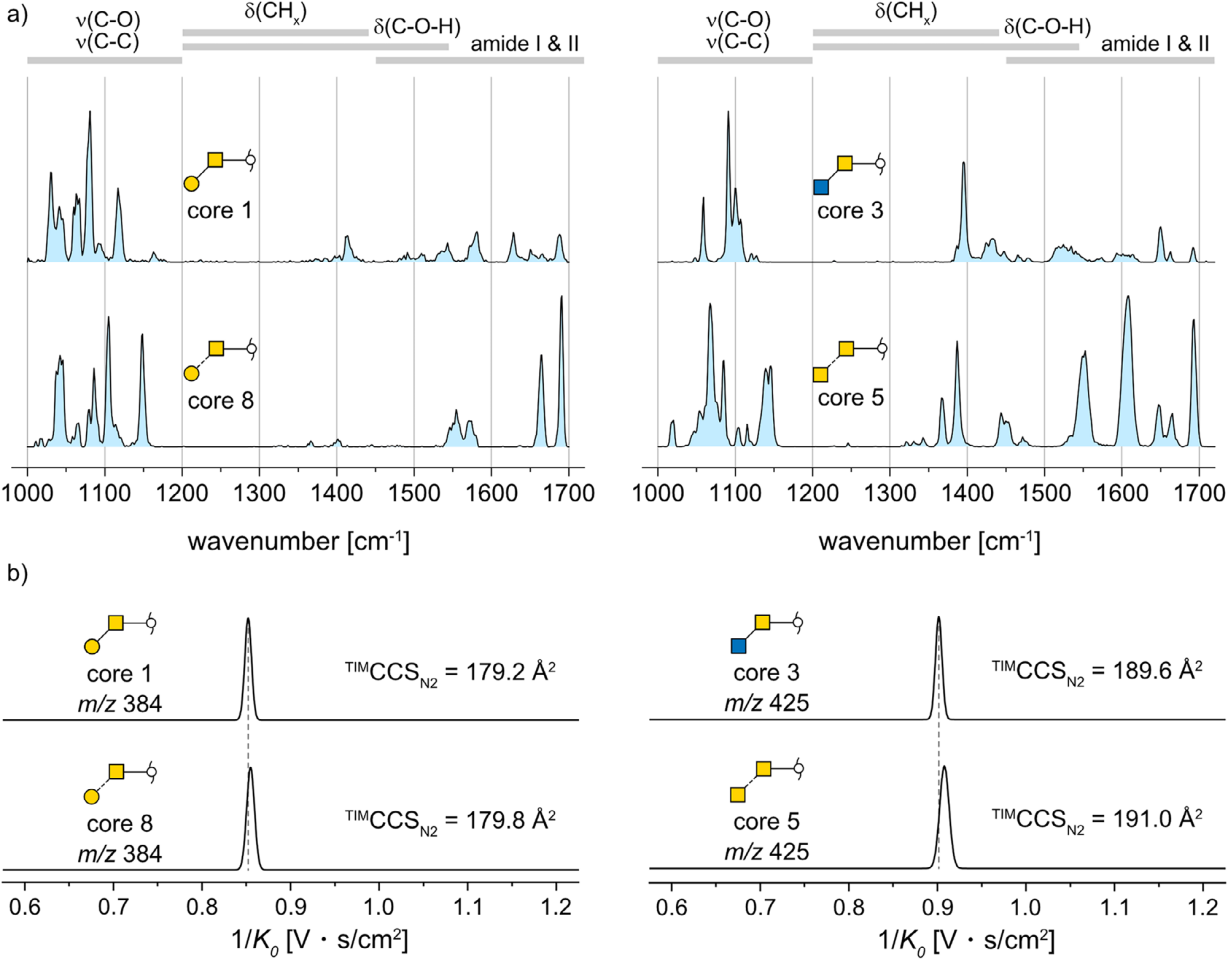
(a) Experimental cryogenic gas‐phase IR spectra of deprotonated core 1,3 5, and 8 alditols recorded in the wavenumber region between 1000 and 1700 cm^−1^. Wavenumber ranges of individual vibrations are depicted as grey bars above the spectra. (b) Extracted ion mobilograms of core 1,3, 5, and 8 alditols. Subtle differences in ion mobility of the isomers are indicated by dashed lines.

We first performed cryogenic gas‐phase IR spectroscopy on the isomeric *O*‐glycan cores. In our experimental setup, helium droplets are used to capture and cool ions to sub‐Kelvin temperatures (0.4 K) reaching their vibrational ground state. Resonant absorption of IR photons causes vibrational heating, which is rapidly dissipated through evaporative cooling leading to an eventual evaporation of the helium matrix or expulsion of the ion, which is monitored by mass spectrometry. Using this set‐up, cryogenic IR spectra of *O*‐glycan cores 1, 3, 5, and 8 alditols were recorded in the range of 1000–1700 cm^−1^ (Figure 2a). The spectra of cores 1 and 8 ([M‐H]^−^, *m/z* 384) show two areas with multiple resolved bands: one in the C–O and C–C stretching region (1000–1200 cm^−1^) and another in the amide I & II region (1400–1700 cm^−1^). Notably, the core 1 alditol features significantly more congestion in the amide I & II region. Additionally, the spectra show distinct patterns in the information dense C–O and C–C stretching regions. Core 8 features two intense bands in this region located at 1105 and 1148 cm^−1^. In the same wavenumber region, almost no absorption was detected for core 1. Although both molecules differ only in the spatial orientation of a single bond, they can be easily distinguished from each other by a simple comparison of their IR signatures.

A similar situation applies to the experimental IR spectra of the core 3 and 5 alditols ([M‐H]^−^, *m/z* 425). Multiple resolved features are observed in the wavenumber regions 1000–1200 cm^−1^ and 1300–1700 cm^−1^. Compared to core 1 and 8, the IR signatures are generally more congested toward higher wavenumbers, which we attribute to the additional *N*‐acetyl group compared to core 3 and 5. The differences between the two IR spectra are even more pronounced as there are multiple bands at 1019, 1067, 1139, 1145, and 1367 cm^−1^ in the spectrum of core 5, which are completely absent in the IR signature of core 3. These distinct differences allow for a simple, unambiguous assignment of core 3 and 5. Furthermore, the comparisons indicate that small segments of the spectra, specifically the wavenumber region between 1000–1200 cm^−1^, are sufficient for identifying the core structures, which reduces analysis time significantly. We conclude that cryogenic IR spectroscopy generates highly diagnostic spectra for *O*‐glycan cores.

Next, we investigated if *O*‐glycan core structures can be separated using ion mobility spectrometry (IMS)‐MS as it has been extensively used to separate challenging isomeric glycans and glycan fragments [28, 29, 30, 31, 32]. To test this, we analyzed the *O*‐glycan core structures using trapped ion mobility spectrometry (TIMS) and travelling wave ion mobility spectrometry (TWIMS) – IMS techniques that have previously demonstrated their ability to separate *O*‐glycan isomers [9]. TIMS mobilograms of core 1 and 8 (Figure 2b) show very subtle differences in mobility and CCS. For core 3 and 5, there is a slightly larger yet still modest difference in mobility. TWIMS experiments (Figure S3) yielded comparable mobility differences and CCS values. The CCSs of the isomer pairs only differ by approximately 1 Å^2^ which is not enough for unambiguous assignment in routine analysis.

### 2.2 Ensembles of Deprotomers Rationalize Complex Vibrational Fingerprints

**FIGURE 3:**
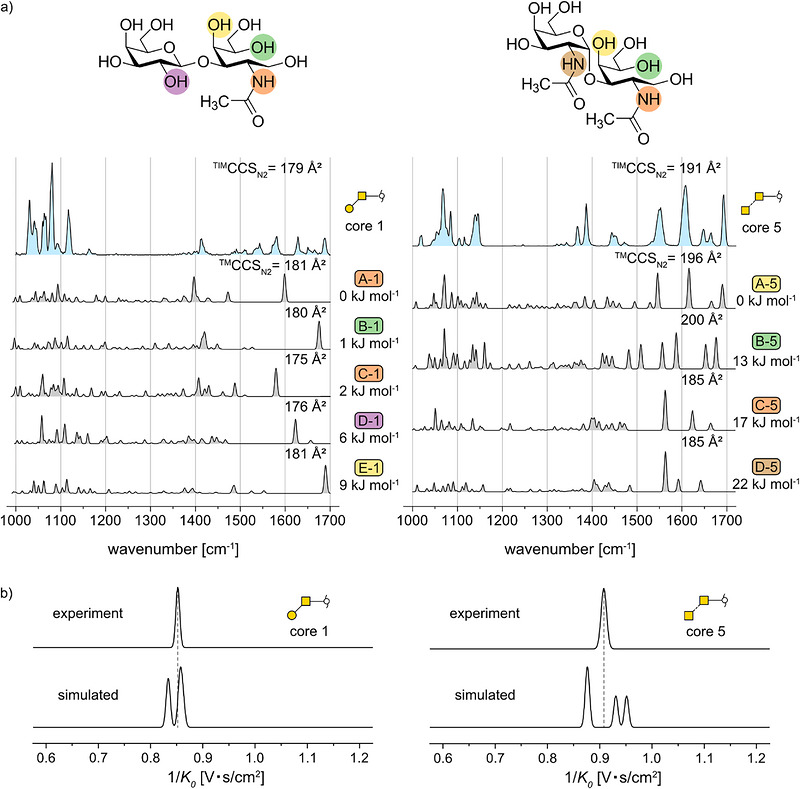
(a) Comparison of experimental (blue trace) and theoretical spectra (gray traces) for deprotonated core 1 and 5 alditols. Relevant deprotonation sites are marked in the structural formulas with different colors and indicated next to theoretical spectra. Relative free energies at 90 K and theoretical CCSs (^TM^CCS_N2_) at 298 K of the individual deprotomers and conformers are indicated. (b) Comparison of experimental and simulated mobilograms for core 1 and 5 alditols. The simulated mobilogram is based on theoretical CCSs of the deprotomers. Differences in ion mobility are highlighted by dashed lines.

Deeper insight into the vibrational bands present in the IR spectra and the three‐dimensional structure of the ions can be obtained by comparing the experimental spectra with those derived from computations. This process is complicated by the multiple deprotonation sites offered by the alditols, hydroxyl, and amide groups. Taking into account the possible deprotonation sites, we computed the low‐energy conformers for each possible *O*‐ and *N*‐deprotomer using DFT calculations and reconstructed theoretical IR spectra from the obtained structures. CAM‐B3LYP was used as the primary functional. To assess the robustness of the calculated relative free energies, additional calculations were performed using B3LYP and PBE0. While minor variations in the ordering are observed, all three functionals place the deprotomers and conformers within a narrow energy window of a few kJ mol^−1^ (Table S2). Therefore, the discussion focuses on the energetic proximity of these structures rather than on assigning a single global minimum. Similar to the experimental spectrum, the computed spectra for the core 1 alditol deprotomers can be roughly divided into three sections: a congested region between 1000 and 1200 cm^−1^ with C–C and C–O stretching vibrations, a sparsely occupied region from 1300 and 1500 cm^−1^ with deformations of C–O–H and C–H moieties, and an amide I & II region between 1550 and 1700 cm^−1^ (Figure 3a). However, the amide region contains only a single, intense vibrational band in all computed spectra whilst the experimental spectrum contains several intense vibrational bands and many more low‐intensity features. This suggests that the experimental spectrum is not fully described by a single harmonic spectrum, but requires contributions from additional structures and/or anharmonic effects. Based on the calculated deprotomers, the core 1 amide group can undergo a redshift from 1620–1700 cm^−1^ to 1550–1600 cm^−1^ when deprotonated (**A‐1** and **C‐1**, orange traces). Hydroxyl deprotomers **B‐1**, **D‐1** and **E‐1** show smaller shifts in the amide region depending on the interactions with the amide NH. We propose that the complex pattern in the amide region is best rationalized by contributions from several low‐energy deprotomers and conformers, while anharmonic effects may additionally contribute to the detailed band pattern. Due to the hydrogen‐bond‐rich and conformationally flexible nature of glycans, anharmonic effects may contribute to discrepancies between calculated and experimental spectra, including band shifts, intensity changes, and peak splittings. However, such effects represent corrections to the harmonic description of a given structural minimum [33, 34]. The close energetic proximity of several deprotomers and conformers, together with the improved agreement obtained when multiple low‐energy structures are considered, indicates that structural heterogeneity contributes to the observed spectral complexity. These structures lie within a narrow energy window and are therefore thermodynamically expected to coexist under the experimental conditions. Accordingly, the experimental spectra are interpreted as arising from low‐energy deprotomer/conformer ensembles, with anharmonic effects contributing to the detailed spectral shape.

For the core 5 alditol, the computed spectra reveal additional IR features in the 1550 to 1700 cm^−1^ region (Figure 3a), which can be attributed to the presence of an additional amide group in a different molecular environment. The amide region does not appear to be significantly more crowded than the core 1 structure, which points towards a smaller distribution of structures. The bands in the amide region do exhibit higher relative intensities compared to those of core 1 alditol. Comparison with the computed spectrum of the lowest‐energy conformer **A‐5** shows a good match. However, the computed spectrum cannot explain the band at 1648 cm^−1^ and the broad asymmetric features at 1550 and 1607 cm^−1^. Although the conformers **B‐5**, **C‐5**, and **D‐5** provide a slightly worse match with the experimental spectrum, they appear to explain the missing spectral features albeit at higher energies. This observation is in alignment with the presence of multiple deprotomers in the probed ensemble of ions for *O*‐glycans, but with structure **A‐5** as the dominant species.

A considerable number of protomers and deprotomers of small molecules were identified by IMS in the past decades [35, 36, 37, 38]. Based on our observations, the question arises as to why none of the investigated isomers show multiple peaks or peak shoulders in the TIMS mobilograms. To understand if the structural differences between the deprotomers would lead to significant changes in the CCS values, we calculated CCS values for each structure. The computed CCS values for the deprotomers and conformers of core 1 and 5 (Figure 3a) show substantial differences, up to 6 Å^2^ and 15 Å^2^ respectively. The experimental CCS values closely align with the computed values for the lowest energy structure. The high resolution of TIMS should clearly resolve the CCS differences among the low‐energy structures. To illustrate this, we simulated mobilograms assuming an equal population of the deprotomers and conformers (Figure 3b). While none of the computed CCS values perfectly align with the experimental results, the experimental CCS values consistently fall within the range of the computed values in both cases. The discrepancy of IMS and IR data points towards a fast interconversion process at room temperature, which is prevented at cryogenic temperatures.

### 2.3 Rapid Gas‐Phase Charge Migration

In aqueous systems, the migration of a proton or proton defect is commonly described by the Grotthuss mechanism, where they are rapidly transferred through a dense hydrogen‐bond network via concerted bond rearrangements [39, 40]. This mechanism allows protons and proton defects to travel much faster through aqueous solutions than other ions. Intramolecular Grotthuss‐type proton transfers in the gas‐phase have been recently described in protonated *p*‐aminobenzoic acid and pyridine solvated by water molecules [41, 42]. In a combined IMS and *ab initio* molecular dynamics study, we revealed first hints for a rapid gas‐phase charge migration in deprotonated human milk oligosaccharides [43]. These results suggest that rapid gas‐phase charge migration in glycans occurs within picoseconds, which would explain the detection of an averaged mobility in IMS measured on the millisecond timescale. However, for desolvated glycan ions in the gas phase, the question arises how intramolecular proton transfers occur without the hydrogen‐bond network of a water shell.

**FIGURE 4:**
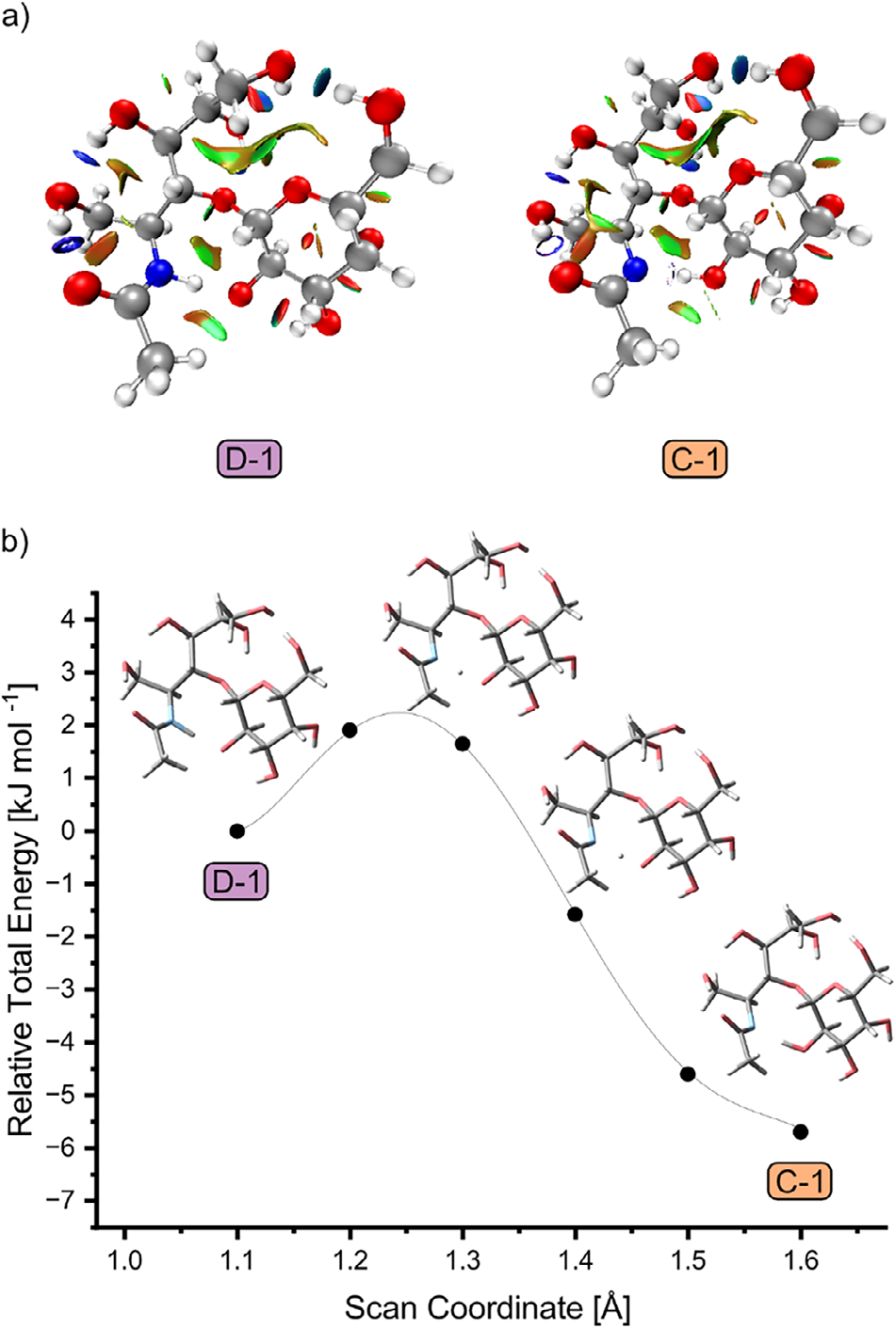
(a) Reduced density gradient isosurface map depicting non‐covalent interactions in the D‐1 and C‐1 deprotomers of core 1 alditol. Blue surfaces indicate strong attractive interactions like hydrogen bonds. (b) Energy barrier for the internal proton transfer process from deprotomer D‐1 to C‐1. Total energies are relative to the D‐1 conformer.

In order to investigate the presence of hydrogen bonds within the *O*‐glycan deprotomers, we performed non‐covalent interaction (NCI) analysis on the low‐energy structures. NCI analysis is described in the following on the examples of **C‐1 and D‐1** (Figure 4a), which share a similar hydrogen‐bond network. Strong non‐covalent interactions like hydrogen bonds are visualized as blue surfaces between the atoms. Green surfaces show weaker attractive forces and red surfaces visualize repulsive forces. In **C‐1** and **D‐1**, hydrogen bonds appear to occur for every hydroxyl group and the amide functional group whenever they are in close spatial proximity. In these networks, the hydrogen atoms seem to be oriented towards close oxygen (or nitrogen) atoms in a similar fashion as observed in water. High levels of internal hydrogen bonding were found for all computed structures, ranging between 5 and 8 internal hydrogen bonds in a single disaccharide alditol (Figures S6–S8).

To further test our hypothesis, we investigated the energetic barriers involved in charge migration processes for glycan deprotomers (Figures S9–S20). Starting from the low‐energy deprotomers, we calculated internal proton transfer events using relaxed potential energy surface scans. For core 1, we computed low energy barriers (<9 kJ mol^−1^) for proton transfers in deprotomers **B‐1**, **C‐1** and **D‐1**. On closer inspection, we found that the structures of **C‐1** and **D‐1** can directly interconvert into each other by the proton transfer process between the amide nitrogen atom and the O2 position of galactose (Figure 4b). According to our calculations, the transition from **D‐1** to **C‐1** involves an energy barrier of approximately 2 kJ mol^−1^ at 0 K, whereas the reverse process from **C‐1** to **D‐1** requires about 8 kJ mol^−1^. **B‐1** transitions into a conformer similar to **D‐1**, which does not fully match its structure. We propose that this charge migration event results in a slightly activated species that can relax to the ground state **D‐1** by bond rotations and rearrangements of the hydrogen bond network. Direct proton transfers within **A‐1** and **E‐1** lead to an increase of 23–36 kJ mol^−1^ at 0 K, which represents energy barriers that may be more challenging to overcome even at ambient temperatures. However, for the possible transfer processes of **E‐1**, we noticed strong rearrangements of the hydrogen‐bond network during the proton transfer. This leads to a distortion of the calculated energy barrier as not only the isolated proton transfer process is considered, but also how the system responds during the transfer, which renders comparison of the proton transfer processes difficult. Preceding or subsequent bond rotations, as well as reorganizations within the hydrogen‐bond network, are extremely challenging to model in *ab initio* calculations. We hypothesize that such processes could enable proton transfers for these deprotomers involving a more complex energy profile.

For core 5, we were unable to discover low‐energy charge migration processes for the lowest energy deprotomer **A‐5**. This absence is in line with the good spectral match provided by this structure. The only direct low‐energy proton transfer event for core 5 structures was found for **B‐5** (Figure S18) with 6 kJ mol^−1^ at 0 K. We attribute the larger energy differences in core 5 to the presence of the second *N*‐acetyl group, which increases the molecular polarity and interferes with the hydrogen‐bond network among the hydroxyl groups.

**FIGURE 5:**
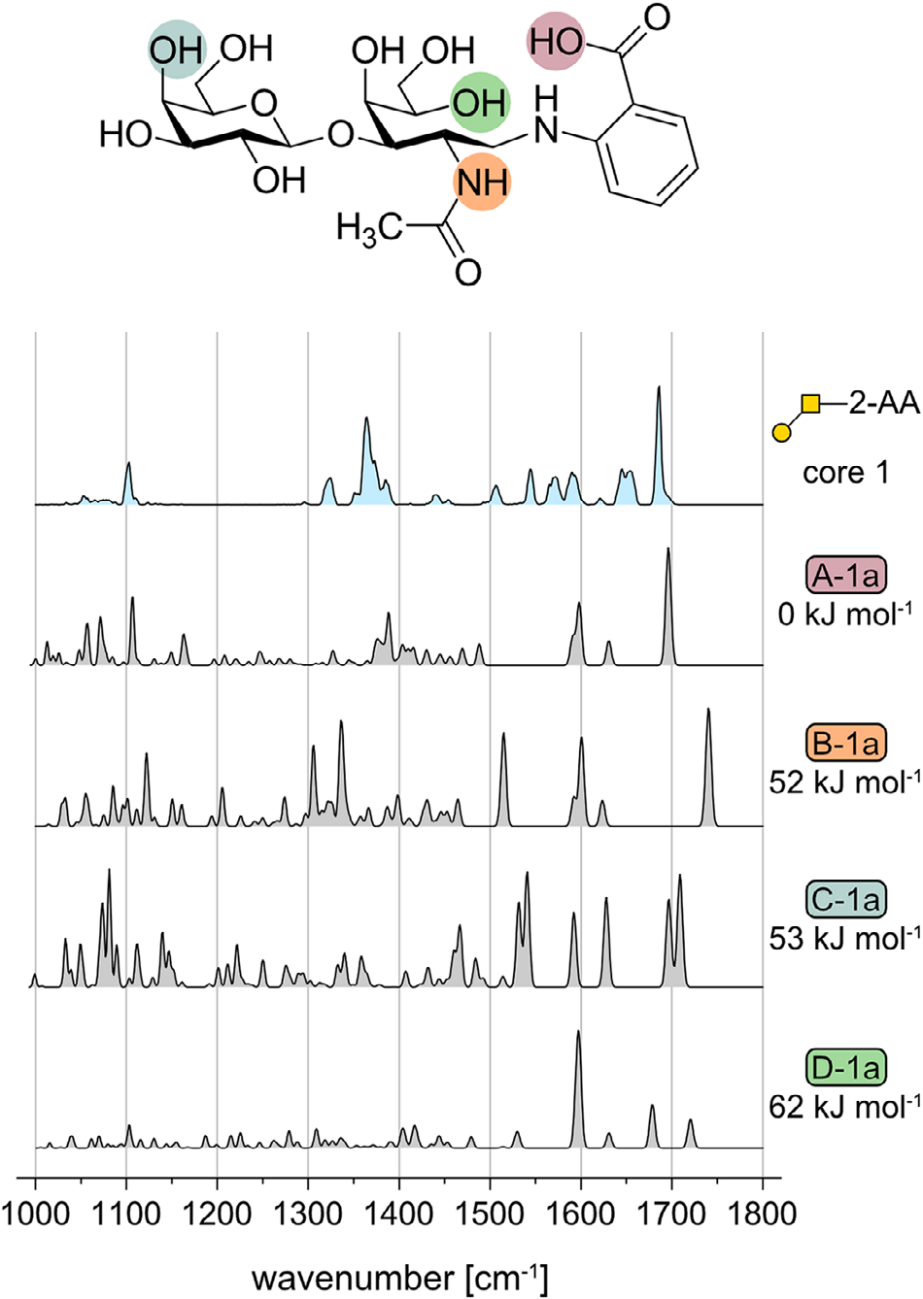
Comparison of experimental (blue trace) and theoretical spectra (gray traces) for deprotonated core 1 labeled with 2‐aminobenzoic acid by reductive amination. Relevant deprotonation sites are marked in the structural formulas with different colors and indicated next to theoretical spectra. Relative free energies at 90 K of the individual deprotomers are indicated.

To directly test whether the presence of a strongly acidic functional group can localize the negative charge and fully suppress charge migration, core 1 was labeled at the reducing end GalNAc using 2‐aminobenzoic acid (2‐AA), which represents a fluorescence label commonly used in glycomics. Comparison with computed IR spectra of different deprotomers (Figure 5) shows that the experimental spectrum of the deprotonated 2‐AA‐labeled core 1 is reproduced exclusively by the carboxylate deprotomer **A‐1a**. Missing vibrational bands can be attributed to other low‐energy conformers (Figure S21) that co‐exist under these experimental conditions. Alternative deprotomers show significant spectral deviations, including additional vibrational bands above 1700 cm^−1^ that are absent from the experimental spectrum. Moreover, these deprotomers were calculated to be at least 50 kJ mol^−1^ higher in relative free energy than deprotomer **A‐1a**. Introduction of the carboxylate leads to strong reduction of OH···OH or OH···O^−^ hydrogen bonds and the resulting hydrogen‐bond network is dominated by interactions of hydroxyl groups with the carboxylate (Figures S22, S23), suggesting a partial shielding of the negative charge of the surrounding hydroxyl groups. These results indicate that the presence of different deprotomers and extent of gas‐phase charge migrations in *O*‐glycans appear to depend strongly on the composition and gas‐phase structure of the molecule. Each system must therefore be assessed individually to evaluate the potential for gas‐phase charge migrations.

Taken together, our gas‐phase experiments and theoretical calculations suggest the presence of intramolecular Grotthuss‐like gas‐phase charge migrations in *O*‐glycan core structures due to the presence of dense hydrogen‐bond networks and the high flexibility of the glycan alditols. This mechanism rationalizes the presence of multiple deprotomers and conformers in the cryogenic IR spectra and explains the absence of complex mobilograms in IMS experiments. This interpretation is further supported by chemical derivatization experiments, which localize the negative charge at a single carboxylate functional group and suppress charge migration, resulting in a well‐defined IR signature.

## 3 Conclusions

In this work, we introduce cryogenic gas‐phase IR spectroscopy to the field of *O*‐glycan analysis. Our results show that IR fingerprints can be used to easily distinguish *O*‐glycan isomers that appear nearly identical in IMS experiments. For practical applications, we identify 1000–1200 cm^−1^ and 1500–1700 cm^−1^ as the spectral regions where the information is most condensed and exhibits the greatest diagnostic potential.

We compared theoretical and experimental IR spectra in order to elucidate the underlying structural information. Surprisingly, we found that no single computed spectrum was able to provide a perfect match with the experimental IR spectra. Instead, the highly diagnostic spectra are best rationalized by contributions from a combination of deprotomers and conformers. We found multiple deprotomers in the probed ensemble of ions at cryogenic temperatures that appear to interconvert in IMS experiments at ambient temperatures. Our *ab initio* calculations explain the observed ensembles by rapid gas‐phase charge migration events, reminiscent of the Grotthuss mechanism in water, in deprotonated glycan structures along internal hydrogen‐bond networks. This interpretation is further supported by experiments in which charge localization was enforced, preventing charge migration and leading to a simplified and well‐defined IR spectrum.

To our knowledge, this establishes for the first time a direct link between IR fingerprints, deprotomer identities, and gas‐phase charge migration in *O*‐glycan alditols and provides a basis for interpreting more complex *O*‐glycan systems. More broadly, our results support a view of gas‐phase *O*‐glycan ions as dynamic ensembles whose structures are shaped by charge position, conformation, and internal hydrogen‐bonding interactions. This fundamental concept connects the two main experimental observation of the study, namely the highly diagnostic cryogenic IR fingerprints and the comparatively simple IMS mobilograms. It further motivates future studies investigating how structurally diverse precursor ensembles may influence tandem MS fragmentation in *O*‐glycomics. We believe that cryogenic gas‐phase IR spectroscopy will become increasingly valuable for the identification of highly complex isomeric structures where tandem MS and IMS approaches struggle, now that the first commercial instruments are coming into reach.

The authors have cited additional references within the Supporting Information [44, 45, 46, 47, 48, 49, 50, 51, 52, 53, 54, 55, 56, 57, 58, 59, 60].

## Conflicts of Interest

The authors declare no conflicts of interest.

## Supporting information




**Supporting File 1**: smll73815‐sup‐0001‐SuppMat.docx.

**Supporting File 2**: smll73815‐sup‐0002‐Data.zip.

## Data Availability

The data that support the findings of this study are available in the supplementary material of this article.

## References

[1] I. Brockhausen , H. Schachter , and P. Stanley , in Essentials of Glycobiology, 2nd Ed. (Eds.: A. Varki , R. D. Cummings , J. D. Esko , et al.,) Chapter 9 (Cold Spring Harbor Laboratory Press, 2009). 20301239

[2] S. K. Linden , P. Sutton , N. G. Karlsson , V. Korolik , and M. A. McGuckin , “Mucins in the Mucosal Barrier to Infection,” Mucosal Immunology 1 (2008): 183–197, 10.1038/mi.2008.5. 19079178 PMC7100821

[3] T. D. Madsen , L. H. Hansen , J. Hintze , et al., “An Atlas of O‐linked Glycosylation on Peptide Hormones Reveals Diverse Biological Roles,” Nature Communications 11 (2020): 4033, 10.1038/s41467-020-17473-1. PMC7441158 32820167

[4] S. L. King , H. J. Joshi , K. T. Schjoldager , et al., “Characterizing the O‐glycosylation Landscape of human Plasma, Platelets, and Endothelial Cells,” Blood Advances 1 (2017): 429–442, 10.1182/bloodadvances.2016002121. 29296958 PMC5738978

[5] A. Varki , R. D. Cummings , M. Aebi , et al., “Symbol Nomenclature for Graphical Representations of Glycans,” Glycobiology 25 (2015): 1323–1324, 10.1093/glycob/cwv091. 26543186 PMC4643639

[6] W. Chai , E. F. Hounsell , G. C. Cashmore , J. R. Rosankiewicz , J. Feeney , and A. M. Lawson , “Characterisation by Mass Spectrometry and 1 H‐NMR of Novel Hexasaccharides among the Acidic O‐Linked Carbohydrate Chains of Bovine Submaxillary Mucin,” European Journal of Biochemistry 207 (1992): 973–980, 10.1111/j.1432-1033.1992.tb17132.x. 1323463

[7] W. G. Chai , E. F. Hounsell , G. C. Cashmore , et al., “Neutral Oligosaccharides of Bovine Submaxillary Mucin,” European Journal of Biochemistry 203 (1992): 257–268, 10.1111/j.1432-1033.1992.tb19855.x. 1730232

[8] M. Safferthal , L. Bechtella , A. Zappe , G. M. Vos , and K. Pagel , “Labeling of Mucin‐Type O ‐Glycans for Quantification Using Liquid Chromatography and Fluorescence Detection,” ACS Measurement Science Au 4 (2024): 223–230, 10.1021/acsmeasuresciau.3c00071. 38645579 PMC11027200

[9] L. Bechtella , J. Chunsheng , K. Fentker , et al., “Ion Mobility‐tandem Mass Spectrometry of Mucin‐type O‐glycans,” Nature Communications 15 (2024): 2611, 10.1038/s41467-024-46825-4. PMC10960840 38521783

[10] A. V. Everest‐Dass , J. L. Abrahams , D. Kolarich , N. H. Packer , and M. P. Campbell , “Structural Feature Ions for Distinguishing N‐ and O‐ Linked Glycan Isomers by LC‐ESI‐IT MS/MS,” Journal of the American Society for Mass Spectrometry 24 (2013): 895–906, 10.1007/s13361-013-0610-4. 23605685

[11] J. Urban , R. Joeres , L. Thomès , and D. Bojar , “Navigating the maze of mass spectra: a machine‐learning guide to identifying diagnostic ions in O‐glycan analysis,” Analytical and Bioanalytical Chemistry 417 (2025): 931–943, 10.1007/s00216-024-05500-9. 39180595 PMC11782297

[12] J. Urban , C. Jin , K. A. Thomsson , et al., “Predicting Glycan Structure from Tandem Mass Spectrometry via Deep Learning,” Nature Methods 21 (2024): 1206–1215, 10.1038/s41592-024-02314-6. 38951670 PMC11239490

[13] C. Jin , D. J. Harvey , W. B. Struwe , and N. G. Karlsson , “Separation of Isomeric O‐ Glycans by Ion Mobility and Liquid Chromatography–Mass Spectrometry,” Analytical Chemistry 91 (2019): 10604–10613, 10.1021/acs.analchem.9b01772. 31298840

[14] E. Mucha , M. Lettow , M. Marianski , et al., “Fucose Migration in Intact Protonated Glycan Ions: a Universal Phenomenon in Mass Spectrometry,” Angewandte Chemie International Edition 57 (2018): 7440–7443, 10.1002/anie.201801418. 29688603

[15] M. Lettow , K. Greis , E. Mucha , et al., “Decoding the Fucose Migration Product during Mass‐Spectrometric Analysis of Blood Group Epitopes,” Angewandte Chemie International Edition 62 (2023): 202302883, 10.1002/anie.202302883. PMC10299593 36939315

[16] B. Moge , B. Schindler , O. Yeni , and I. Compagnon , Angewandte Chemie International Edition 62 (2023): 202300538. 10.1002/anie.202300538 36825496

[17] N. Geue , M. Safferthal , and K. Pagel , “Collision‐Induced Fragmentation of Oligosaccharides: Mechanistic Insights for Mass Spectrometry‐Based Glycomics,” Angewandte Chemie International Edition 64 (2025): 202511591, 10.1002/anie.202511591. PMC12338385 40631944

[18] E. Mucha , A. I. González Flórez , M. Marianski , et al., “Glycan Fingerprinting via Cold‐Ion Infrared Spectroscopy,” Angewandte Chemie International Edition 56 (2017): 11248–11251, 10.1002/anie.201702896. 28513924

[19] N. Khanal , C. Masellis , M. Z. Kamrath , D. E. Clemmer , and T. R. Rizzo , “Cryogenic IR Spectroscopy Combined with Ion Mobility Spectrometry for the Analysis of human Milk Oligosaccharides,” The Analyst 143 (2018): 1846–1852, 10.1039/C8AN00230D. 29541730 PMC5902639

[20] A. H. Abikhodr , A. Ben Faleh , S. Warnke , V. Yatsyna , and T. R. Rizzo , “Identification of human Milk Oligosaccharide Positional Isomers by Combining IMS‐CID‐IMS and Cryogenic IR Spectroscopy,” The Analyst 148 (2023): 2277–2282, 10.1039/D3AN00407D. 37098888 PMC10186583

[21] A. H. Abikhodr , S. Warnke , A. Ben Faleh , and T. R. Rizzo , “Combining Liquid Chromatography and Cryogenic IR Spectroscopy in Real Time for the Analysis of Oligosaccharides,” Analytical Chemistry 96 (2024): 1462–1467, 10.1021/acs.analchem.3c03578. 38211954 PMC10831784

[22] P. Bansal , A. Ben Faleh , S. Warnke , and T. R. Rizzo , “Identification of N ‐glycan Positional Isomers by Combining IMS and Vibrational Fingerprinting of Structurally Determinant CID Fragments,” The Analyst 147 (2022): 704–711, 10.1039/D1AN01861B. 35079754 PMC8842669

[23] A. Ben Faleh , S. Warnke , P. Bansal , R. P. Pellegrinelli , I. Dyukova , and T. R. Rizzo , “Identification of Mobility‐Resolved N ‐Glycan Isomers,” Analytical Chemistry 94 (2022): 10101–10108, 10.1021/acs.analchem.2c01181. 35797429 PMC9310030

[24] X. Song , H. Ju , Y. Lasanajak , M. R. Kudelka , D. F. Smith , and R. D. Cummings , “Oxidative Release of Natural Glycans for Functional Glycomics,” Nature Methods 13 (2016): 528–534, 10.1038/nmeth.3861. 27135973 PMC4887297

[25] G. M. Vos , J. Weber , I. R. Sweet , K. C. Hooijschuur , J. Sastre Toraño , and G.‐J. Boons , “Oxidative Release of O ‐Glycans under Neutral Conditions for Analysis of Glycoconjugates Having Base‐Sensitive Substituents,” Analytical Chemistry 95 (2023): 8825–8833, 10.1021/acs.analchem.3c00127. 37259796 PMC10267892

[26] P. H. Jensen , N. G. Karlsson , D. Kolarich , and N. H. Packer , “Structural Analysis of N‐ and O‐glycans Released from Glycoproteins,” Nature Protocols 7 (2012): 1299–1310, 10.1038/nprot.2012.063. 22678433

[27] H. Wilkinson and R. Saldova , “Current Methods for the Characterization of O ‐Glycans,” Journal of Proteome Research 19 (2020): 3890–3905, 10.1021/acs.jproteome.0c00435. 32893643

[28] J. Hofmann , W. B. Struwe , C. A. Scarff , J. H. Scrivens , D. J. Harvey , and K. Pagel , “Estimating Collision Cross Sections of Negatively Charged N‐ Glycans Using Traveling Wave Ion Mobility‐Mass Spectrometry,” Analytical Chemistry 86 (2014): 10789–10795, 10.1021/ac5028353. 25268221

[29] J. Hofmann , H. S. Hahm , P. H. Seeberger , and K. Pagel , “Identification of Carbohydrate Anomers Using Ion Mobility–mass Spectrometry,” Nature 526 (2015): 241–244, 10.1038/nature15388. 26416727

[30] J. Hofmann , A. Stuckmann , M. Crispin , D. J. Harvey , K. Pagel , and W. B. Struwe , “Identification of Lewis and Blood Group Carbohydrate Epitopes by Ion Mobility‐Tandem‐Mass Spectrometry Fingerprinting,” Analytical Chemistry 89 (2017): 2318–2325, 10.1021/acs.analchem.6b03853. 28192913

[31] R. L. Miller , S. E. Guimond , R. Schwörer , et al., “Shotgun Ion Mobility Mass Spectrometry Sequencing of Heparan Sulfate Saccharides,” Nature Communications 11 (2020): 1481, 10.1038/s41467-020-15284-y. PMC7083916 32198425

[32] G. M. Vos , K. C. Hooijschuur , Z. Li , et al., “Sialic Acid O‐acetylation Patterns and Glycosidic Linkage Type Determination by Ion Mobility‐mass Spectrometry,” Nature Communications 14 (2023): 6795, 10.1038/s41467-023-42575-x. PMC10600165 37880209

[33] V. Barone , “Anharmonic vibrational properties by a fully automated second‐order perturbative approach,” The Journal of Chemical Physics 122, no. 1 (2005): 014108, 10.1063/1.1824881. 15638643

[34] J. Bloino and V. Barone , “A second‐order perturbation theory route to vibrational averages and transition properties of molecules: General formulation and application to infrared and vibrational circular dichroism spectroscopies,” The Journal of Chemical Physics 136, no. 12 (2012): 124108, 10.1063/1.3695210. 22462836

[35] S. Warnke , J. Seo , J. Boschmans , et al., “Protomers of Benzocaine: Solvent and Permittivity Dependence,” Journal of the American Chemical Society 137 (2015): 4236–4242. 25760934 10.1021/jacs.5b01338

[36] P. M. Lalli , B. A. Iglesias , H. E. Toma , et al., “Protomers: Formation, Separation and Characterization via Travelling Wave Ion Mobility Mass Spectrometry,” Journal of Mass Spectrometry 47 (2012): 712–719, 10.1002/jms.2999. 22707163

[37] H. Xia and A. B. Attygalle , “Transformation of the Gas‐Phase Favored O‐Protomer of p‐Aminobenzoic Acid to Its Unfavored N‐Protomer by Ion Activation in the Presence of Water Vapor: A n Ion‐Mobility Mass Spectrometry Study,” Journal of Mass Spectrometry 53 (2018): 353–360, 10.1002/jms.4066. 29377420

[38] H. Xia and A. B. Attygalle , “Effect of Electrospray Ionization Source Conditions on the Tautomer Distribution of Deprotonated p ‐Hydroxybenzoic Acid in the Gas Phase,” Analytical Chemistry 88 (2016): 6035–6043, 10.1021/acs.analchem.6b01230. 27164186

[39] C. J. T. de Grotthuss , Annales De Chimie 58 (1806): 54–73.

[40] D. Marx , “Proton Transfer 200 Years after von Grotthuss: Insights from Ab Initio Simulations,” Chemphyschem 7 (2006): 1848–1870, 10.1002/cphc.200600128. 16929553

[41] K. Hirata , K. Akasaka , O. Dopfer , S.‐I. Ishiuchi , and M. Fujii , “Transition from Vehicle to Grotthuss Proton Transfer in a Nanosized Flask: Cryogenic Ion Spectroscopy of Protonated p ‐aminobenzoic Acid Solvated with D_2_O,” Chemical Science 15 (2024): 2725–2730, 10.1039/D3SC05455A. 38404372 PMC10882521

[42] Y. Okura , G. D. Santis , K. Hirata , et al., “Switching of Protonation Sites in Hydrated Nicotine via a Grotthuss Mechanism,” Journal of the American Chemical Society 146 (2024): 3023–3030, 10.1021/jacs.3c08922. 38261007

[43] W. B. Struwe , C. Baldauf , J. Hofmann , P. M. Rudd , and K. Pagel , “Ion Mobility Separation of Deprotonated Oligosaccharide Isomers—Evidence for Gas‐phase Charge Migration,” Chemical Communications 52 (2016): 12353–12356, 10.1039/C6CC06247D. 27711324

[44] A. I. González Flórez , E. Mucha , D.‐S. Ahn , et al., Angewandte Chemie International Edition 55 (2016): 3295–3299. 26847383 10.1002/anie.201510983 PMC4770441

[45] U. Even , “The Even‐Lavie Valve as a Source for High Intensity Supersonic Beam,” EPJ Techniques and Instrumentation 2 (2015): 17, 10.1140/epjti/s40485-015-0027-5.

[46] W. Schöllkopf , S. Gewinner , and H. Junkes , Proceedings of SPIE 9512 (2015): 95121L.

[47] M. Götze , L. Polewski , L. Bechtella , K. Pagel , et al., Journal of the American Society for Mass Spectrometry 34 (2023): 2403–2406. 37602654 10.1021/jasms.3c00214 PMC10557379

[48] K. Pagel and D. J. Harvey , “Ion Mobility–Mass Spectrometry of Complex Carbohydrates: Collision Cross Sections of Sodiated N‐linked Glycans,” Analytical Chemistry 85 (2013): 5138–5145, 10.1021/ac400403d. 23621517

[49] P. Pracht , F. Bohle , and S. Grimme , “Automated Exploration of the Low‐energy Chemical Space with Fast Quantum Chemical Methods,” Physical Chemistry Chemical Physics 22 (2020): 7169–7192, 10.1039/C9CP06869D. 32073075

[50] C. Bannwarth , S. Ehlert , and S. Grimme , “GFN_2‐x_TB—An Accurate and Broadly Parametrized Self‐Consistent Tight‐Binding Quantum Chemical Method with Multipole Electrostatics and Density‐Dependent Dispersion Contributions,” Journal of Chemical Theory and Computation 15 (2019): 1652–1671, 10.1021/acs.jctc.8b01176. 30741547

[51] R. Krishnan , J. S. Binkley , R. Seeger , and J. A. Pople , “Self‐consistent Molecular Orbital Methods. XX. A Basis Set for Correlated Wave Functions,” The Journal of Chemical Physics 72 (1980): 650–654, 10.1063/1.438955.

[52] T. Yanai , D. P. Tew , and N. C. Handy , “A New Hybrid Exchange–correlation Functional Using the Coulomb‐attenuating Method (CAM‐B3LYP),” Chemical Physics Letters 393 (2004): 51–57, 10.1016/j.cplett.2004.06.011.

[53] S. Grimme , S. Ehrlich , and L. Goerigk , “Effect of the Damping Function in Dispersion Corrected Density Functional Theory,” Journal of Computational Chemistry 32 (2011): 1456–1465, 10.1002/jcc.21759. 21370243

[54] G. W. T. M. J. Frisch , H. B. Schlegel , G. E. Scuseria , et al., Revision A.03 ed., Gaussian Inc, Wallingford CT, 2016.

[55] U. C. Singh and P. A. Kollman , “An Approach to Computing Electrostatic Charges for Molecules,” Journal of Computational Chemistry 5 (1984): 129–145, 10.1002/jcc.540050204.

[56] M. F. Mesleh , J. M. Hunter , A. A. Shvartsburg , G. C. Schatz , and M. F. Jarrold , “Structural Information from Ion Mobility Measurements: Effects of the Long‐Range Potential,” The Journal of Physical Chemistry 100 (1996): 16082–16086, 10.1021/jp961623v.

[57] L. Zanotto , G. Heerdt , P. C. T. Souza , G. Araujo , and M. S. Skaf , “High Performance Collision Cross Section Calculation—HPCCS,” Journal of Computational Chemistry 39 (2018): 1675–1681, 10.1002/jcc.25199. 29498071

[58] T. Lu , “A Comprehensive Electron Wavefunction Analysis Toolbox for Chemists, Multiwfn,” The Journal of Chemical Physics 161 (2024): 082503, 10.1063/5.0216272. 39189657

[59] T. Lu and F. Chen , “Multiwfn: a Multifunctional Wavefunction Analyzer,” Journal of Computational Chemistry 33 (2012): 580–592, 10.1002/jcc.22885. 22162017

[60] W. Humphrey , A. Dalke , and K. Schulten , “VMD: Visual Molecular Dynamics,” Journal of Molecular Graphics 14 (1996): 33–38, 10.1016/0263-7855(96)00018-5. 8744570

[61] L. Bennett , B. Melchers , and B. Proppe , Curta: A General‐purpose High‐Performance Computer at ZEDAT, Freie Universität Berlin, https://refubium.fu‐berlin.de/handle/fub188/26993?locale‐attribute=en, (May 2025).

