# Supplementary material for: Cryogenic Infrared Spectroscopy Unmasks Gas‐Phase Charge Migration in Mucin‐Type O‐Glycans
Source: Small. 2026 May 15;22(38):e00077. doi: 10.1002/smll.202600077 (PMC13351509; doi:10.1002/smll.202600077)
Supplement: Supplementary file 1 — Supporting File 1: smll73815‐sup‐0001‐SuppMat.docx. [file SMLL-22-e00077-s001.docx]

Supporting Information

Cryogenic Infrared Spectroscopy Unmasks Gas-Phase Charge Migration in Mucin-Type *O*-Glycans

Marc Safferthal,^ab^ Gurpur Rakesh D. Prabhu,^ab^ América Y. Torres-Boy,^b^ Jerome Riedel,^ab^ Leïla Bechtella,^ab^ Wesley Pietsch,^ab^ Gerard Meijer,^b^ Gert von Helden,^b^ Gaël M. Vos,^ab^ Kevin Pagel*^ab^

^a^ Department of Biology, Chemistry, Pharmacy, Freie Universität Berlin, Altensteinstraße 23a, 14195 Berlin, Germany

^b^ Fritz Haber Institute of the Max Planck Society, Faradayweg 4-6, 14195 Berlin, Germany

^*^ Corresponding author. E-mail: kevin.pagel@fu-berlin.de

**Experimental Section**

**Materials**

*O*‑glycan standards (Galβ1,3GalNAc, Galα1,3GalNAc, GlcNAcβ1,3GalNAc, and GalNAcα1,3GalNAc) were purchased from Biosynth (Switzerland). HPLC-grade acetonitrile (ACN), and methanol (MeOH) were purchased from VWR Chemicals (USA). Ammonium acetate, sodium borohydride, acetic acid, 2-aminobenzoic acid (2-AA), Dowex 50W-X8 cation exchange resin, and dextran Mw 1000 were obtained from Sigma-Aldrich (USA). All chemicals were used as received without further purification.

**Reduction** **of Free Reducing End Glycans**

*O*-glycan standards (100 µg) were dissolved in 1 M aqueous sodium borohydride solution (120 µL) and incubated at 50°C for 3h under mild agitation. The reaction was quenched by the addition of acetic acid (12 µL) and desalted using 400 mg Dowex 50WX8 cation exchange beads. The resin was washed three times with MeOH (1 mL), conditioned with 1 M HCl (1 mL), and washed with methanol (1 mL) and H_2_O (1 mL) prior to use. The glycans were loaded on the resin, eluted with H_2_O (2 x 1 mL), and dried *via* SpeedVac. Borates were removed by addition and evaporation of MeOH (750 µL).

**2-AA Labeling of Free Reducing End Glycans**

Labeling solution was prepared by dissolving sodium cyanoborohydride (6 mg) and 2-AA (6 mg) in 30% acetic acid in DMSO (100 µL). *O*-glycan standards (100 µg) were dissolved in labeling solution (35 µL) and incubated at 65°C for 2h. The reaction mixture was dried *via* SpeedVac and purified by HPLC-FLD.

**Purification of 2- AA-Labeled *O*-Glycans Using LC-FLD**

LC-FLD purification was performed using an Azura HPLC system (Knauer, Germany) equipped with a Dionex UltiMate 3000 fluorescence detector (Thermo Fisher Scientific). Wavelengths of the fluorescence detector were set to λ_ex_ = 320 nm and λ_em_ = 420 nm. After labeling, glycans were separated using an Acquity UPLC BEH amide column (130 Å, 1.7 μm, 2.1 mm × 150 mm, Waters, U.K.) with a flow rate of 0.4 ml/min. The column oven was set to 60°C. Solvent A was 50 mM ammonium formate adjusted to pH 4.4. Solvent B was acetonitrile. Glycans were separated using an isocratic gradient at 90% B for 5 min, followed by a linear gradient of 90–60% B from 5 to 25 min. Fractions were collected manually.

**Cryogenic Gas-Phase IR Spectroscopy in Helium Droplets**

Cryogenic gas-phase IR spectroscopy was performed on a custom‑built instrument, which has been described in detail previously.^[1, 2]^ Glycan alditols were dissolved in 50 mM ammonium acetate in H_2_O:MeOH (1:1). The samples are ionized *via* nano-ESI. The ions of interest are *m/z*-selected in a quadrupole and accumulated and thermalized to 90 K in a hexapole ion trap. Superfluid helium droplets, generated by an Even-Lavie-valve,^[3]^ pick up the ions, cool them to the helium equilibrium temperature (0.4 K), and transfer the encapsulated analytes out of the trap to the interaction region. Here, the analytes encounter the IR photons generated by the Fritz Haber Institute free-electron laser (FHI-FEL).^[4]^ Sequential absorption and relaxation events from multiple resonant IR photons leads to evaporation of the helium matrix and subsequently to the detection of the ions by a time‑of‑flight mass analyzer. IR spectra are obtained by plotting the ion count as a function of the wavenumber. The ion count was divided by the photon fluence of the IR macropulse (assuming a constant absorption cross section) to account for fluctuations of the laser during the measurements. Mass spectra of the deprotonated *O*-glycan alditols before and after *m/z*‑selection can be found in the Figure S1.

**Ion Mobility Spectrometry**

TIM-MS was performed on a timsTOF Pro spectrometer (Bruker, Bremen, Germany), equipped with an in-house 3D-printed offline nano-ESI source.^[5]^ Glycan alditols were dissolved in 50 mM ammonium acetate in H_2_O:MeOH (1:1). For each measurement, 5 μL of sample was introduced in a Pt/Pd-coated glass capillary emitter prepared in-house and ionized in negative ion mode using nano-ESI. The instrument parameters were set as follows: capillary voltage 1.0 kV, end plate offset −0.5 kV, dry source temperature 150 °C, D1 = 150 V, D2 = 30 V, D3 = −100 V, D4 = −100 V, D5 = 0 V, and D6 = −10 V. The TIMS separation was performed in N_2_. Ions reversed mobility 1/*K_0_* was scanned between 0.31 V·s/cm^2^ and 1.39 V·s/cm^2^. The ramp time was set to 1000 ms. The transfer time was fixed to 2 ms. The data was acquired with Compass otofControl (version 6.2, Bruker) and the mobilograms were processed using DataAnalysis (version 4.0, Bruker). In order to obtain estimated ^TIM^CCS_N2_, data treatment, and mobility calibration were performed using a dextran ladder 1k as described previously.^[6]^

_PGC_LC-TWIM-MS was performed using a SYNAPT G2-S spectrometer (Waters, Manchester, U.K.) equipped with an Acquity UPLC system. Glycan alditols were dissolved in 1 mL H_2_O and 5 μL were injected. Glycan alditols were separated using a 100 × 2.1 mm I.D. PGC column of 5 μm particle size (Hypercarb, Thermo Scientific, U.S.A.). Glycan alditols were eluted using a linear gradient from 0 to 40% ACN in 10 mM NH_4_HCO_3_ over 40 min at a flow rate of 150 μL/min. Glycans were ionized in ESI negative ion mode with a capillary voltage of 2.2 kV and a source temperature of 150 °C. The mass range was set to *m/z* 50-3500. *O*-glycan mobilities were recorded in nitrogen (N_2_) using an IMS wave velocity of 450 m/s, and an IMS wave height of 40 V. The data was acquired and processed using MassLynx (version 2.0.7, Waters). In order to obtain estimated ^TWIMS^CCS_N2_, data treatment, and mobility calibration were performed using a dextran ladder 1k as described previously.^[7, 8]^

**Computational Methods**

The conformational space of the *O*-glycan alditols was explored using CREST^[9]^ with the semiempirical method GFN2-xTB^[10]^ and default settings. Geometry optimizations, harmonic frequency calculations and relaxed potential energy surface scans were performed at the CAM-B3LYP+D3BJ/6-311+G(d,p)^[11-13]^ level of theory in Gaussian 16.^[14]^ All frequencies were scaled by an empirical factor of 0.965. Relative free energies of the conformers were calculated for the ion trap temperature of 90 K and the indicated energies are relative to the lowest‑energy conformer. Partial atomic charges were calculated according to the Merz‑Singh‑Kollman scheme^[15]^ under the constraint of reproducing the overall molecular dipole moment in Gaussian 16. The CCSs of conformers were computed using the trajectory method^[16]^ as implemented in the software HPCCS.^[17]^ CCSs were calculated in nitrogen at 298 K. Reduced density gradient (RDG) isosurface maps and scatter plots for non-covalent interaction (NCI) analysis were generated and visualized using Multiwfn^[18, 19]^ (version 3.8) and VMD^[20]^ (version 1.9.3). Simulated TIMS mobilograms were generated using a custom Python script written in-house, which can be found in a separate document “convolution_simulation.py”. The script models a sum of Gaussian peaks in 1/K_0_ space, defined by center positions, widths, and relative intensities. The peak width was modeled inferred from the apparent FHWM in the experimental mobilograms.


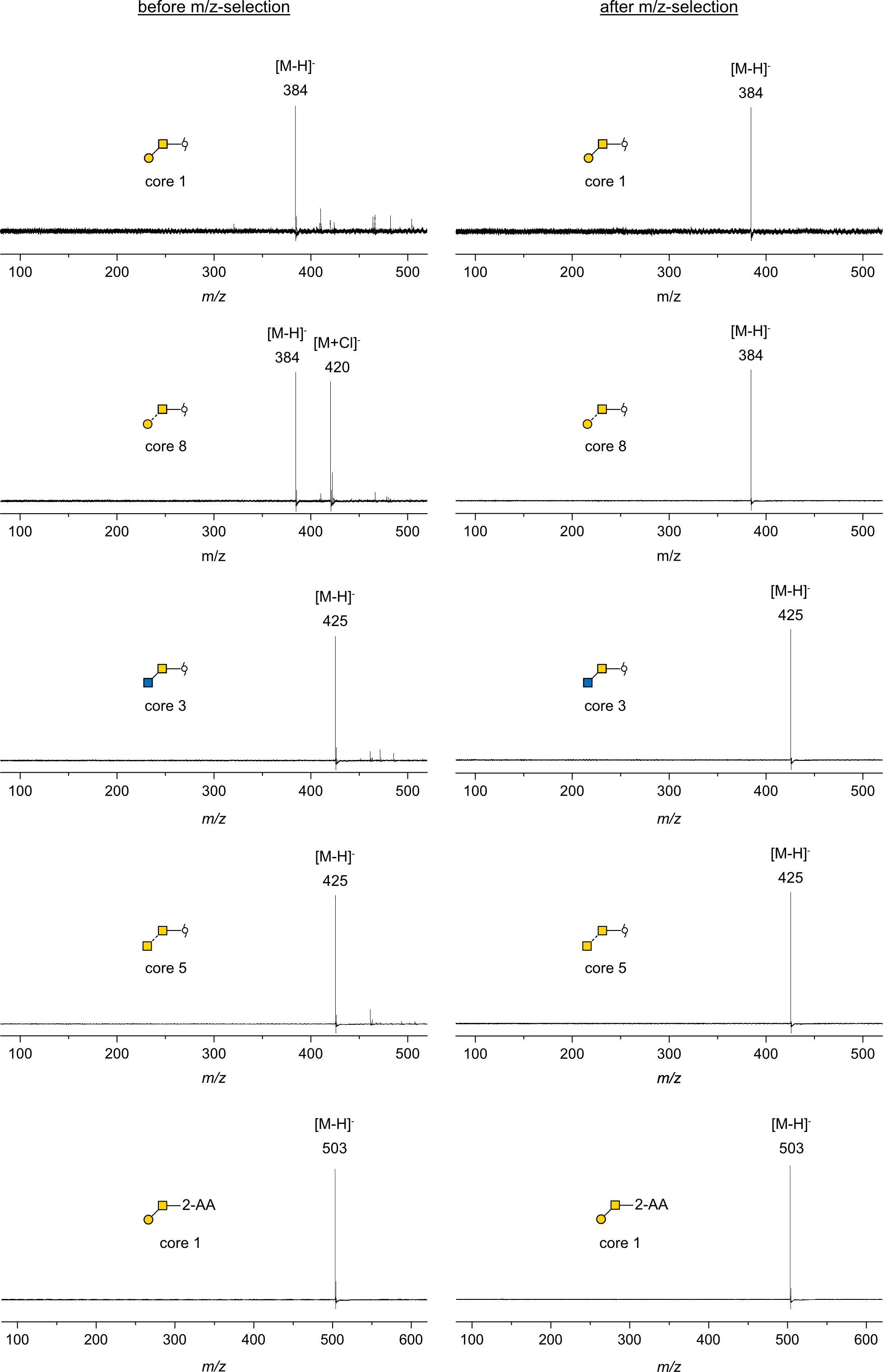


Figure S1. Mass spectra of deprotonated *O*-glycan core alditols and 2-AA-labeled core 1 before (left) and after (right) m/z-selection.


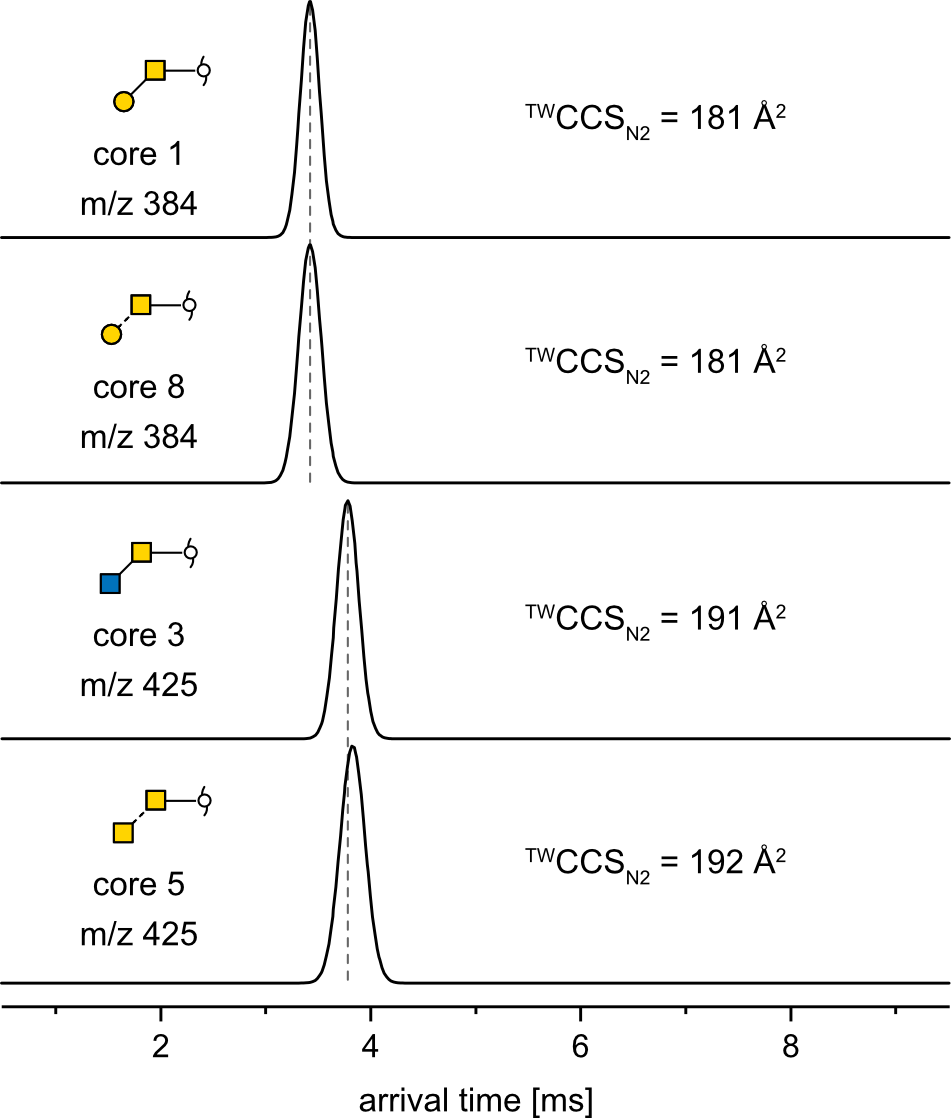


Figure S2. Extracted ion mobilograms (EIMs) of deprotonated *O*-glycan alditols from TWIM-MS experiments.


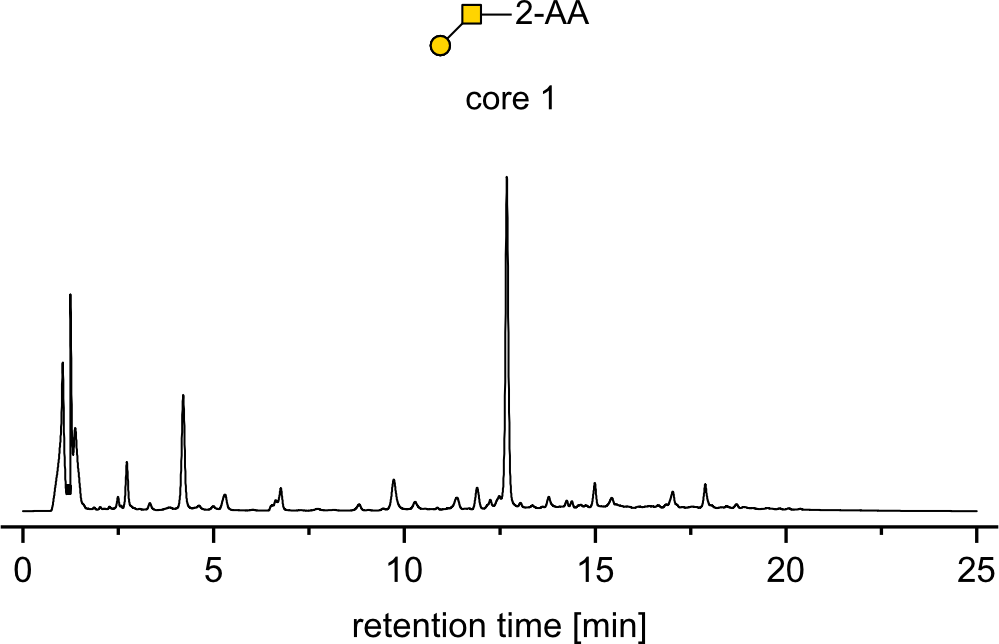


Figure S3. Chromatogram of 2-AA-labeled core 1 recorded using LC-FLD.


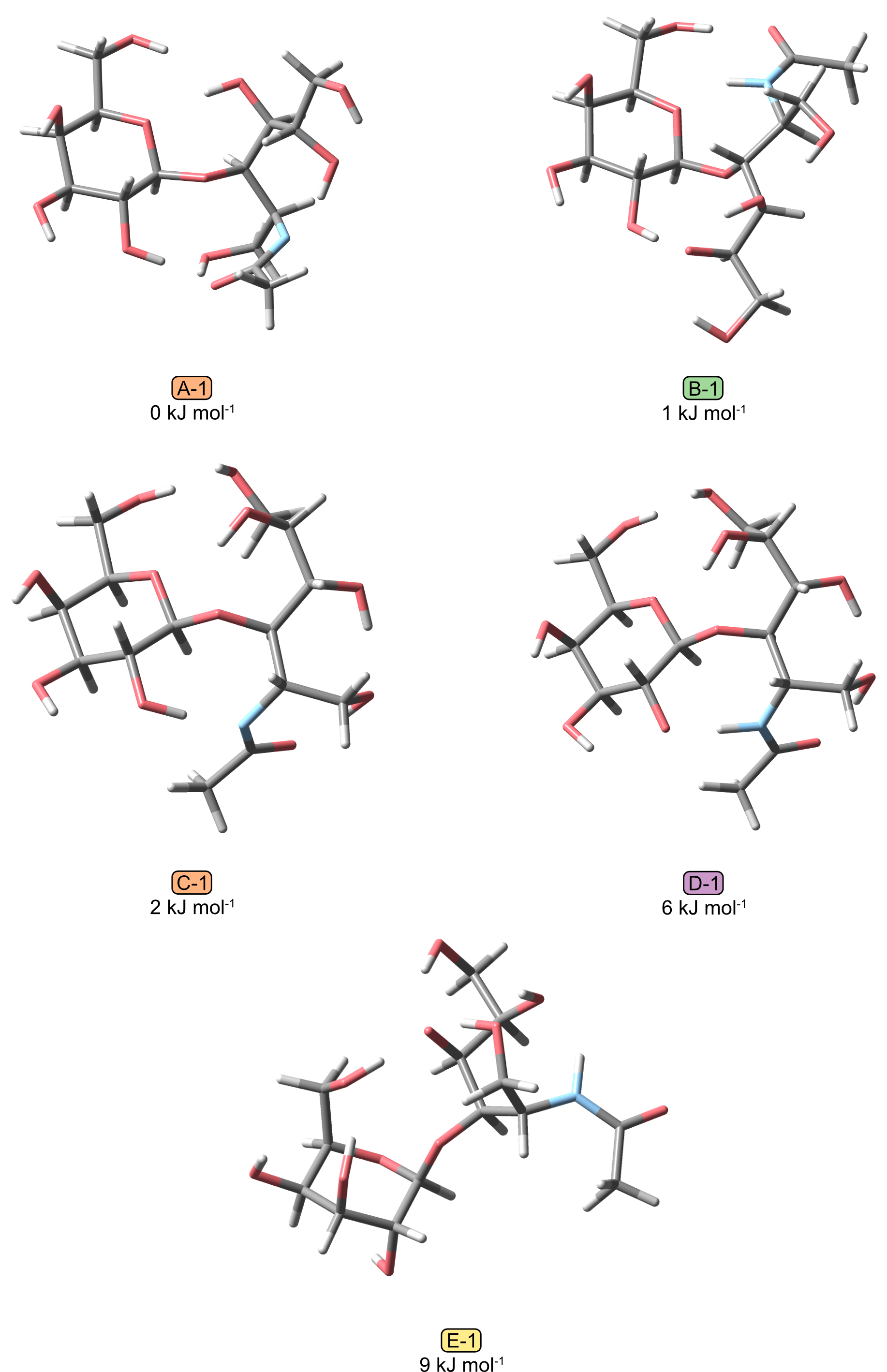


Figure S4. Computed structures of low-energy conformers and deprotomers for deprotonated core 1 alditol. Relative free energies at 90 K are indicated.


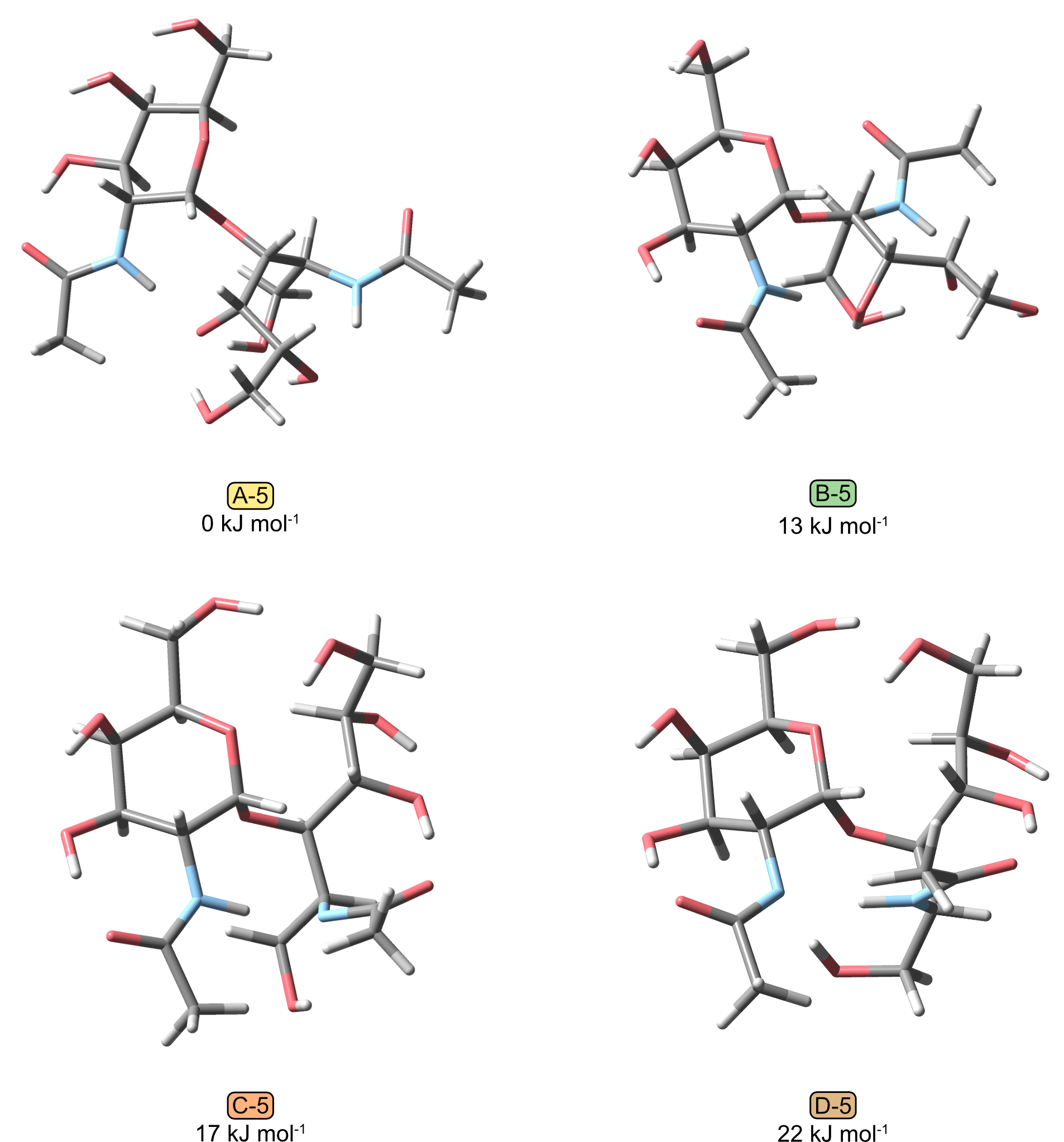


Figure S5. Computed structures of low-energy conformers and deprotomers for deprotonated core 5 alditol. Relative free energies at 90 K are indicated.

**Table S1.** Relative zero-point corrected total energies (ΔE), free energies at 90 K (ΔF) and CCS values in nitrogen (^TM^CCS_N2_) of computed structures reoptimized at the CAM-B3LYP+D3BJ/6-311+G(d,p) level of theory.

| structure | core | ΔE | ΔF | ^TM^CCS_N2_ |
| --- | --- | --- | --- | --- |
| **A-1** | 1 | 0.0 kJ mol^-1^ | 0.0 kJ mol-1 | 181 Å |
| **B-1** | 1 | 0.6 kJ mol^-1^ | 1.3 kJ mol-1 | 180 Å |
| **C-1** | 1 | 0.0 kJ mol^-1^ | 1.5 kJ mol-1 | 175 Å |
| **D-1** | 1 | 4.8 kJ mol^-1^ | 6.5 kJ mol-1 | 176 Å |
| **E-1** | 1 | 8.2 kJ mol^-1^ | 8.5 kJ mol-1 | 181 Å |
| **A-5** | 5 | 0.0 kJ mol^-1^ | 0.0 kJ mol^-1^ | 196 Å |
| **B-5** | 5 | 12.7 kJ mol^-1^ | 12.7 kJ mol^-1^ | 200 Å |
| **C-5** | 5 | 15.4 kJ mol^-1^ | 16.9 kJ mol^-1^ | 185 Å |
| **D-5** | 5 | 20.1 kJ mol^-1^ | 22.2 kJ mol^-1^ | 185 Å |

Table S2. Relative free energies at 90 K (ΔF) of computed structures reoptimized at the CAM-B3LYP+D3BJ/6-311+G(d,p), B3LYP+D3BJ/6-311+G(d,p) and PBE0+D3BJ/6-311+G(d,p) levels of theory.

| structure | core | CAM-B3LYP (ΔF) | PBE0 (ΔF) | B3LYP (ΔF) |
| --- | --- | --- | --- | --- |
| **A-1** | 1 | 0.0 kJ mol-1 | 1.6 kJ mol-1 | 0.0 kJ mol-1 |
| **B-1** | 1 | 1.3 kJ mol-1 | 0.8 kJ mol-1 | 1.4 kJ mol-1 |
| **C-1** | 1 | 1.5 kJ mol-1 | 0.0 kJ mol-1 | 0.3 kJ mol-1 |
| **D-1** | 1 | 6.5 kJ mol-1 | 3.6 kJ mol-1 | 5.2 kJ mol-1 |
| **E-1** | 1 | 8.5 kJ mol-1 | 5.0 kJ mol-1 | 2.7 kJ mol-1 |

**
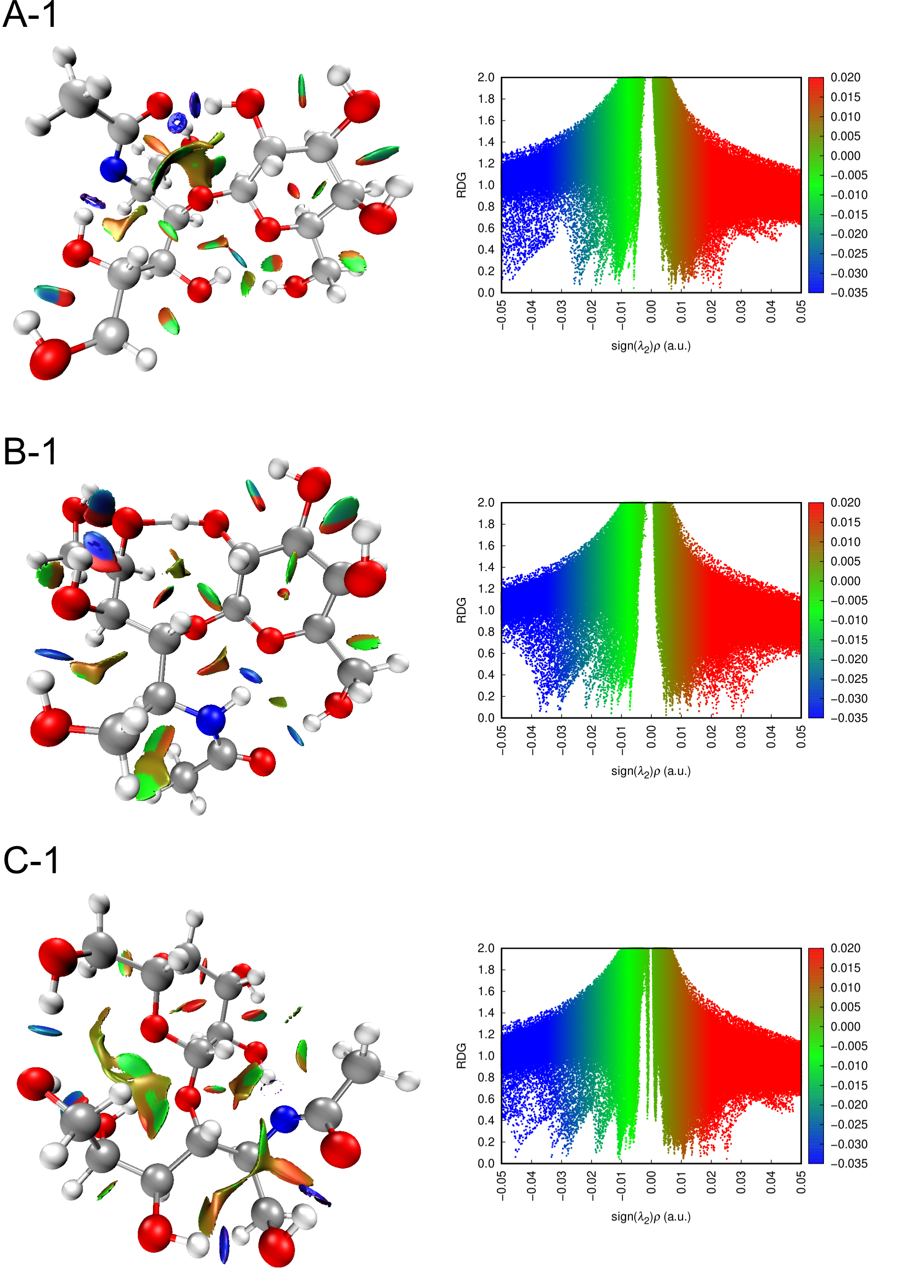
**

Figure S6. Non-covalent interaction analysis of lowest-energy confomers of core 1 deprotomers. Reduced density gradient isosurface map (left) and scatter plot (right) for conformer A-1 (top), B-1 (middle), and C-1 (bottom).


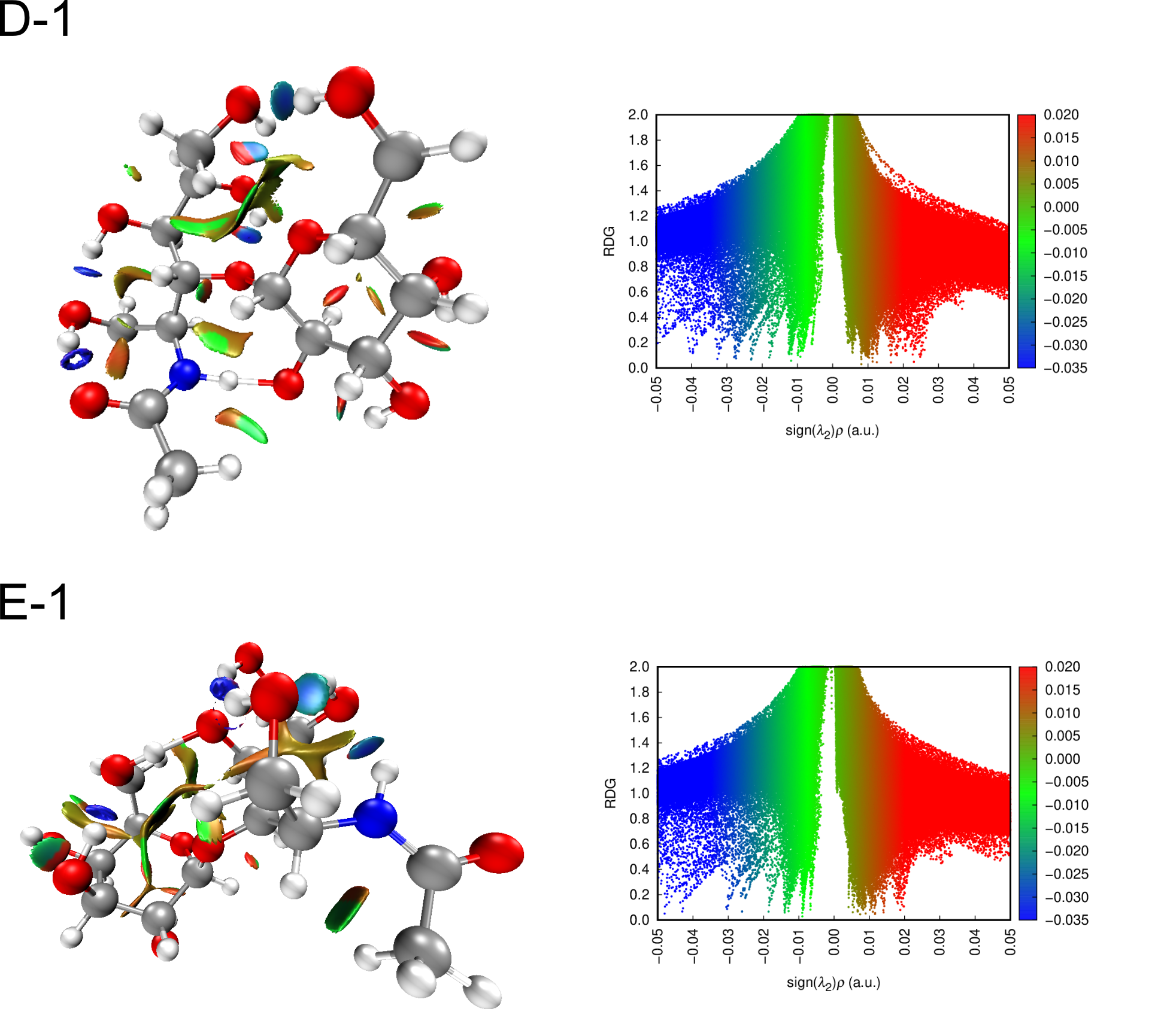


Figure S7. Non-covalent interaction analysis of lowest-energy confomers of core 1 deprotomers. Reduced density gradient isosurface map (left) and scatter plot (right) for conformer D-1 (top) and E-1 (bottom).


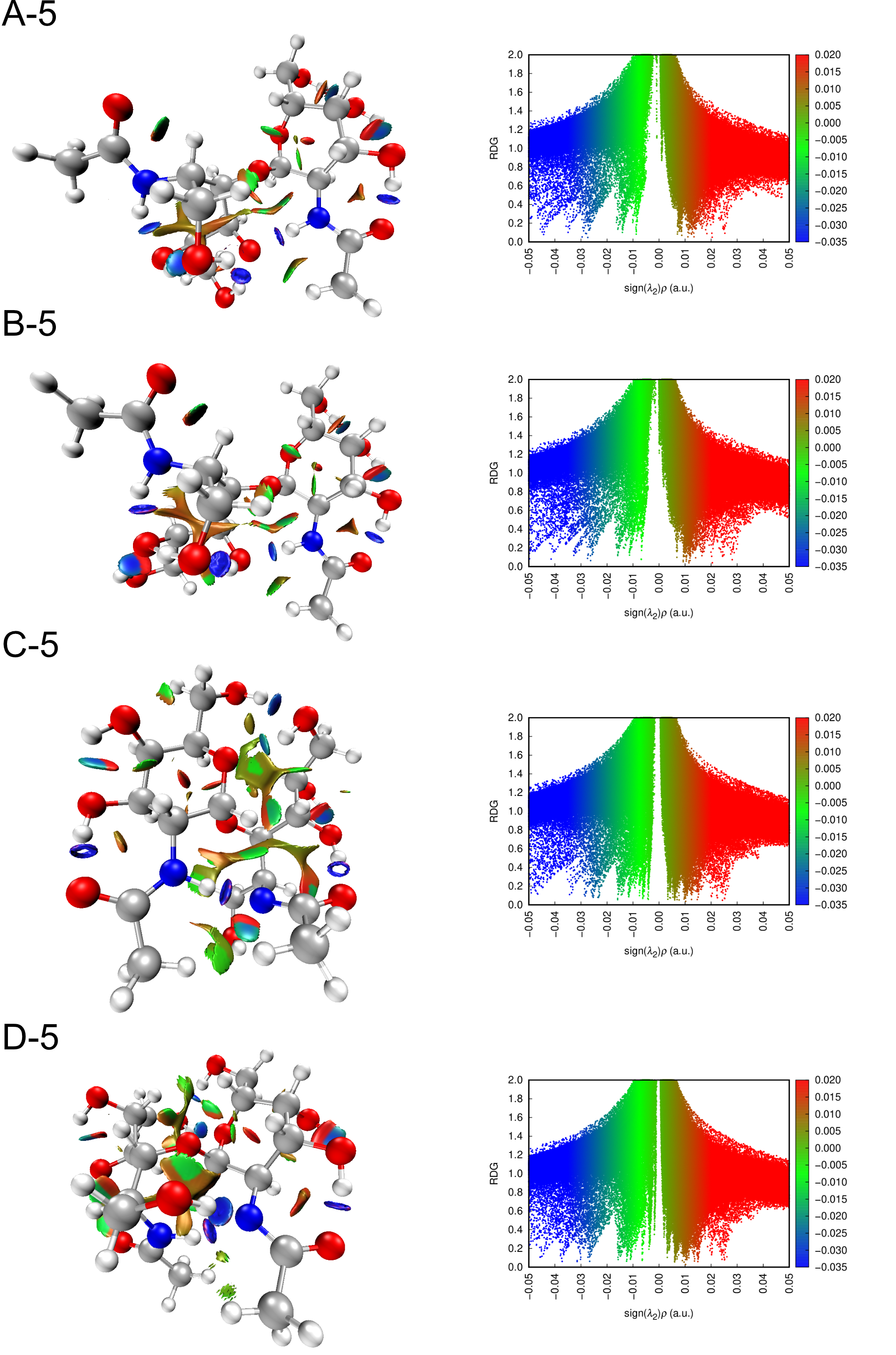


Figure S8. Non-covalent interaction analysis of lowest-energy confomers of core 1 deprotomers. Reduced density gradient isosurface map (left) and scatter plot (right) for conformer A-5, B-5, C-5 and D-5.


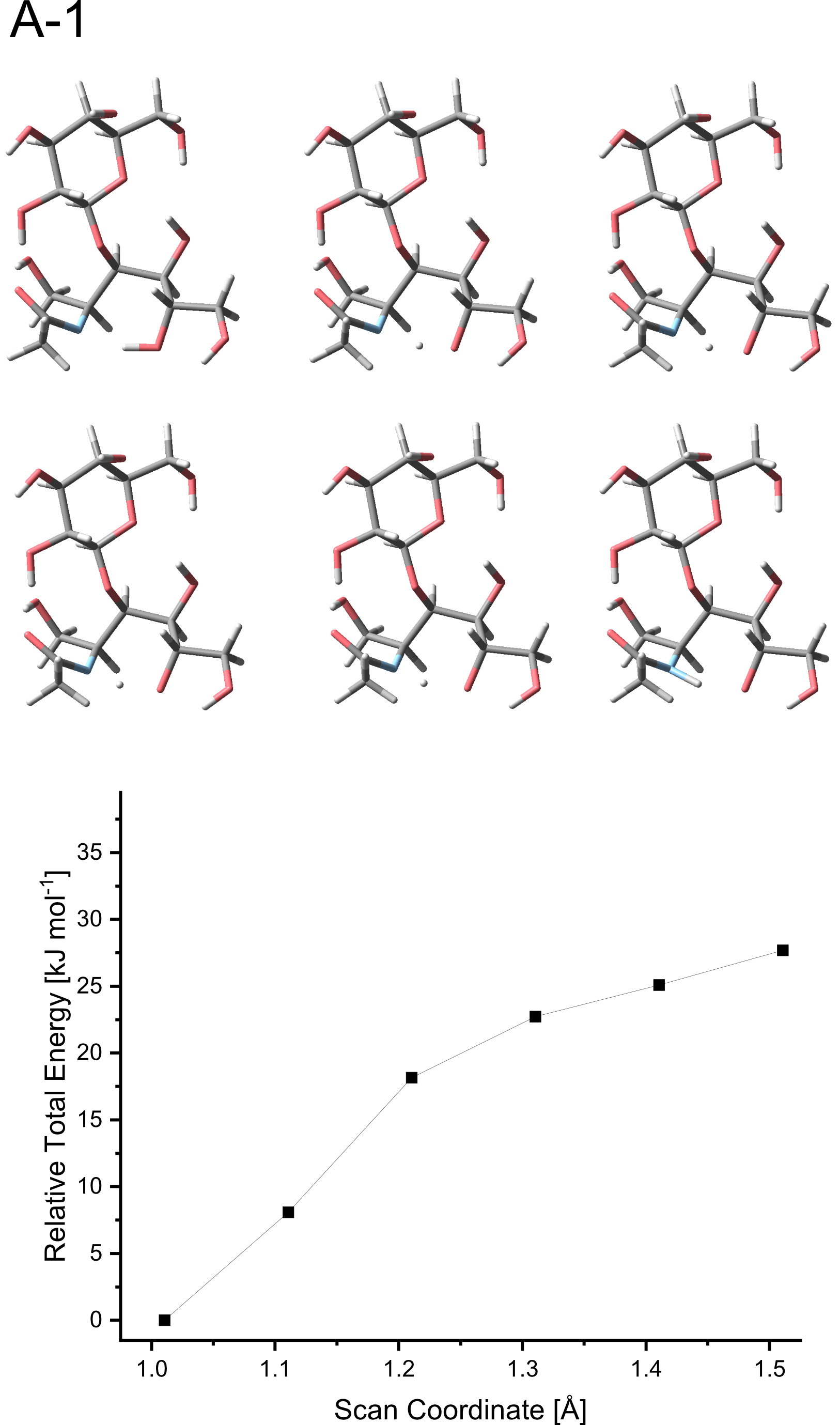


Figure S9. Relaxed potential energy surface scans for the internal proton transfer process in conformer A-1. Total energies are relative to the initial structure.


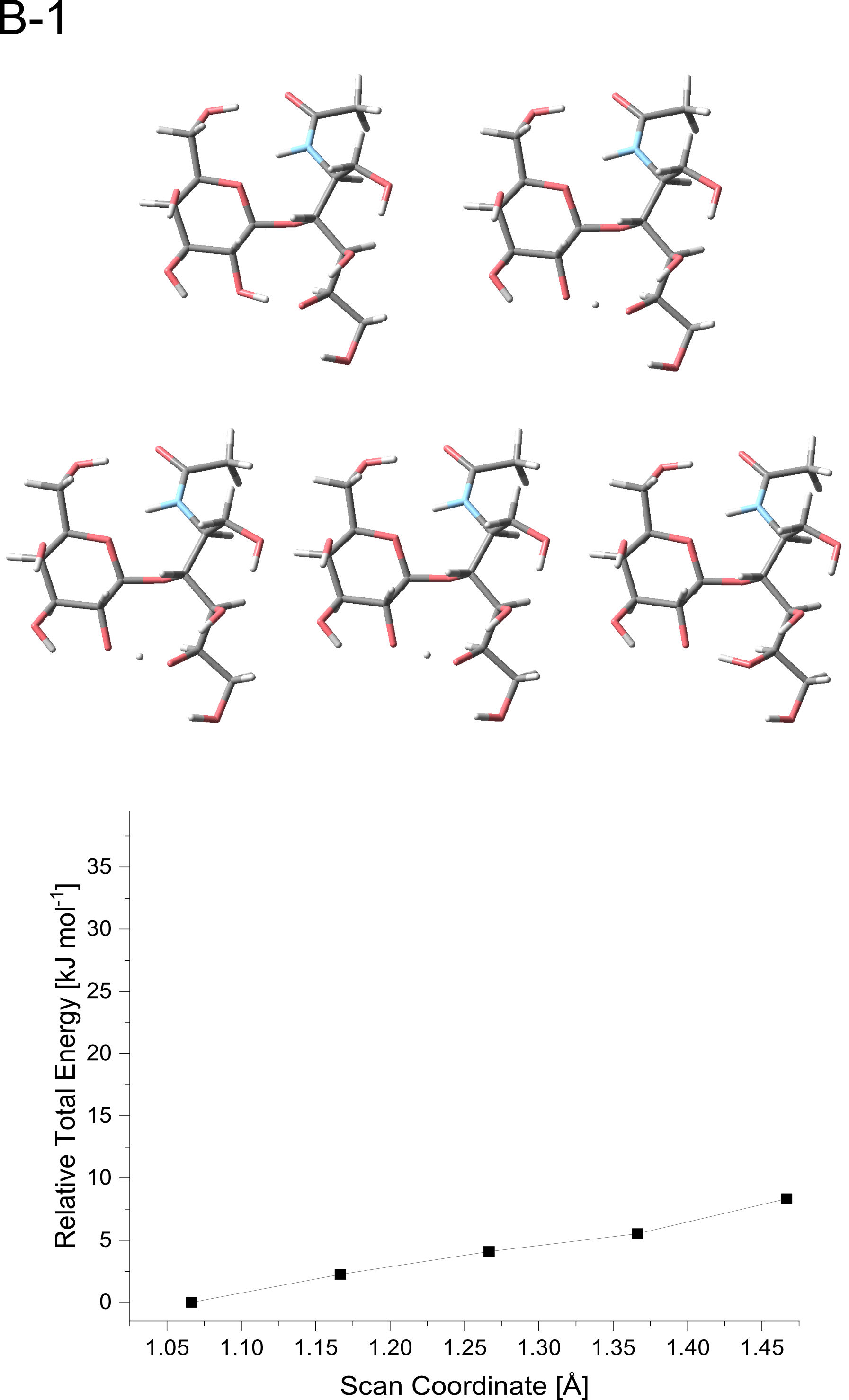


Figure S10. Relaxed potential energy surface scans for the internal proton transfer process in conformer B-1. Total energies are relative to the initial structure.


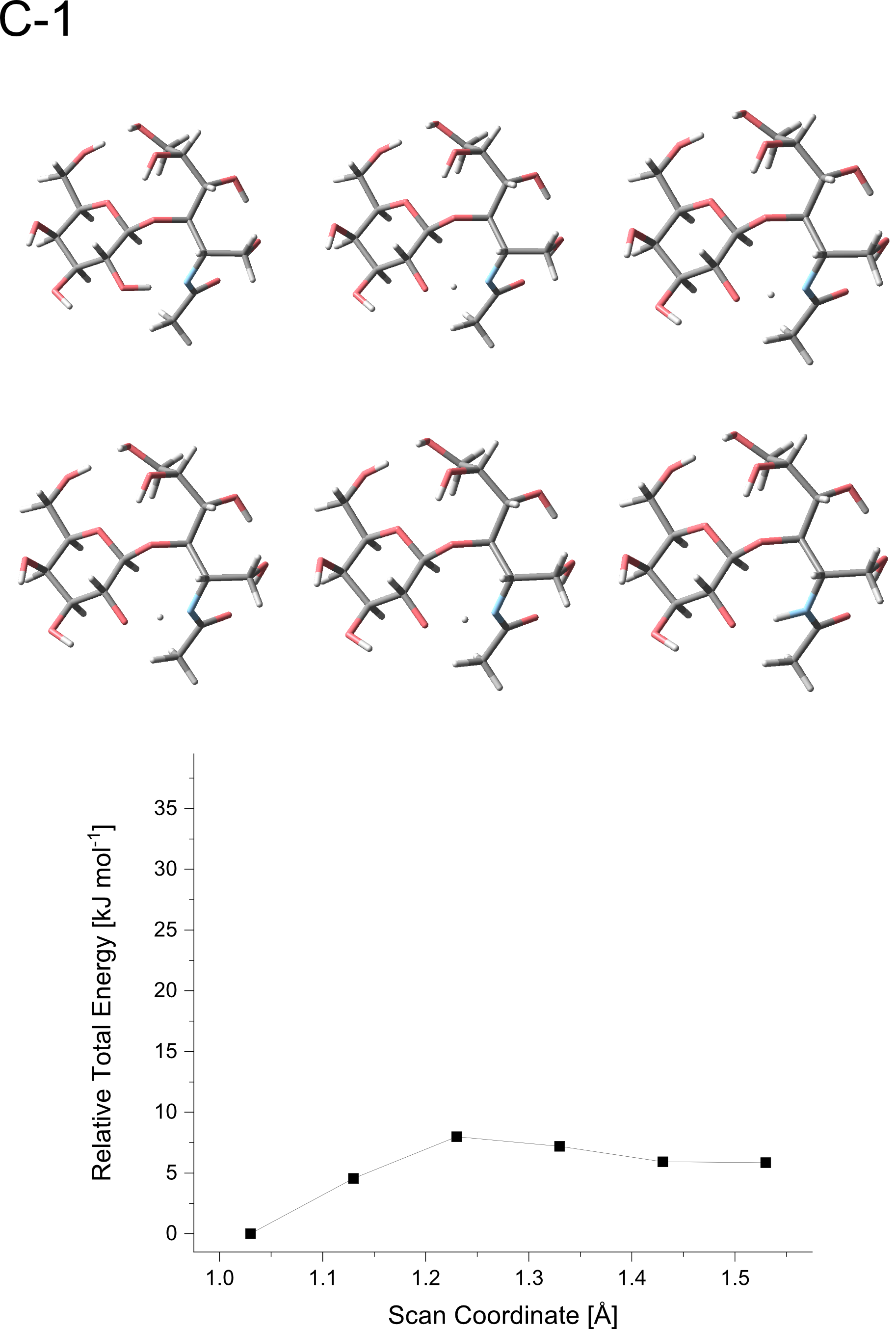


Figure S11. Relaxed potential energy surface scans for the internal proton transfer process in conformer C-1. Total energies are relative to the initial structure.


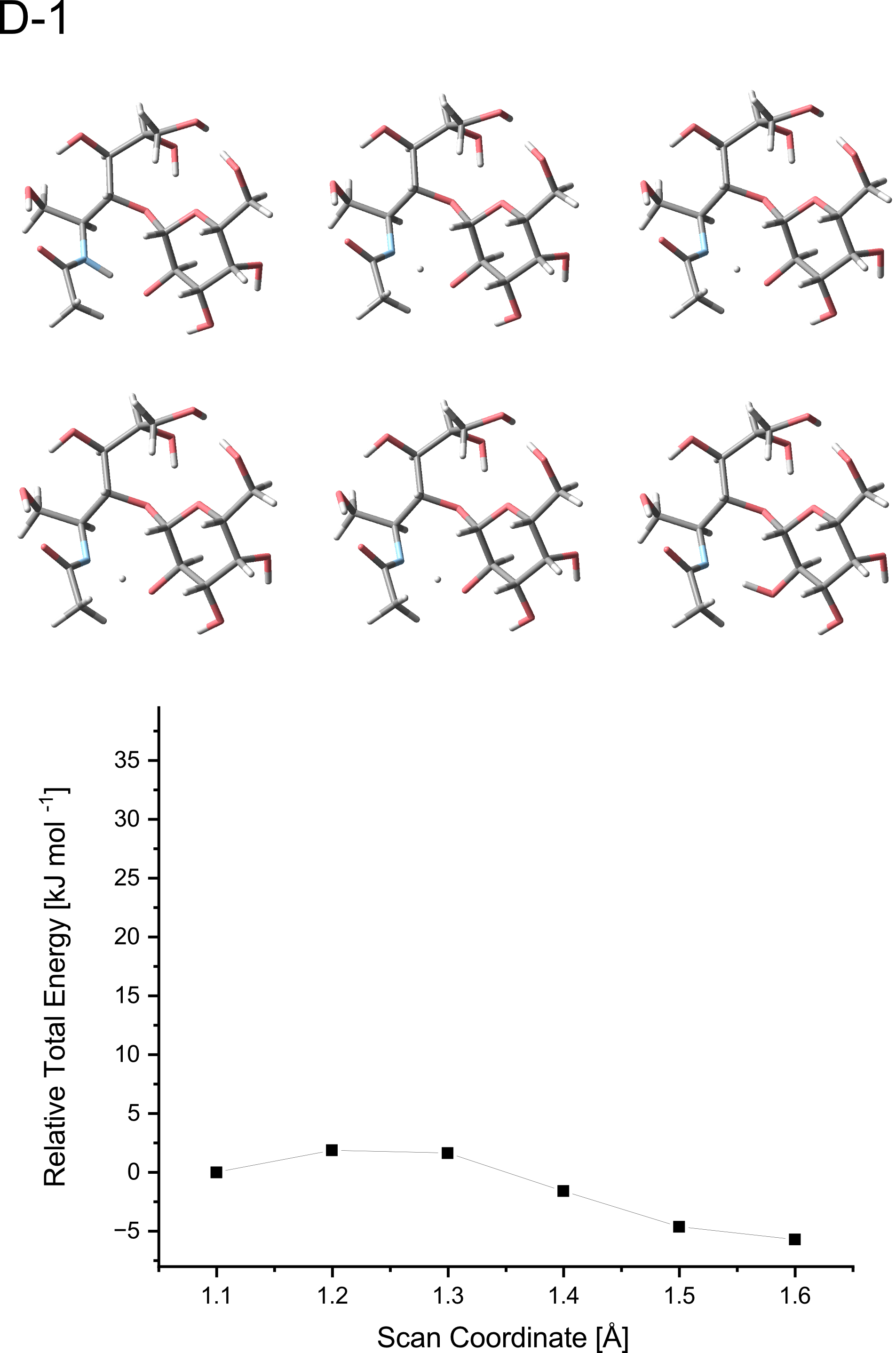


Figure S12. Relaxed potential energy surface scans for the internal proton transfer process in conformer D-1. Total energies are relative to the initial structure.

**
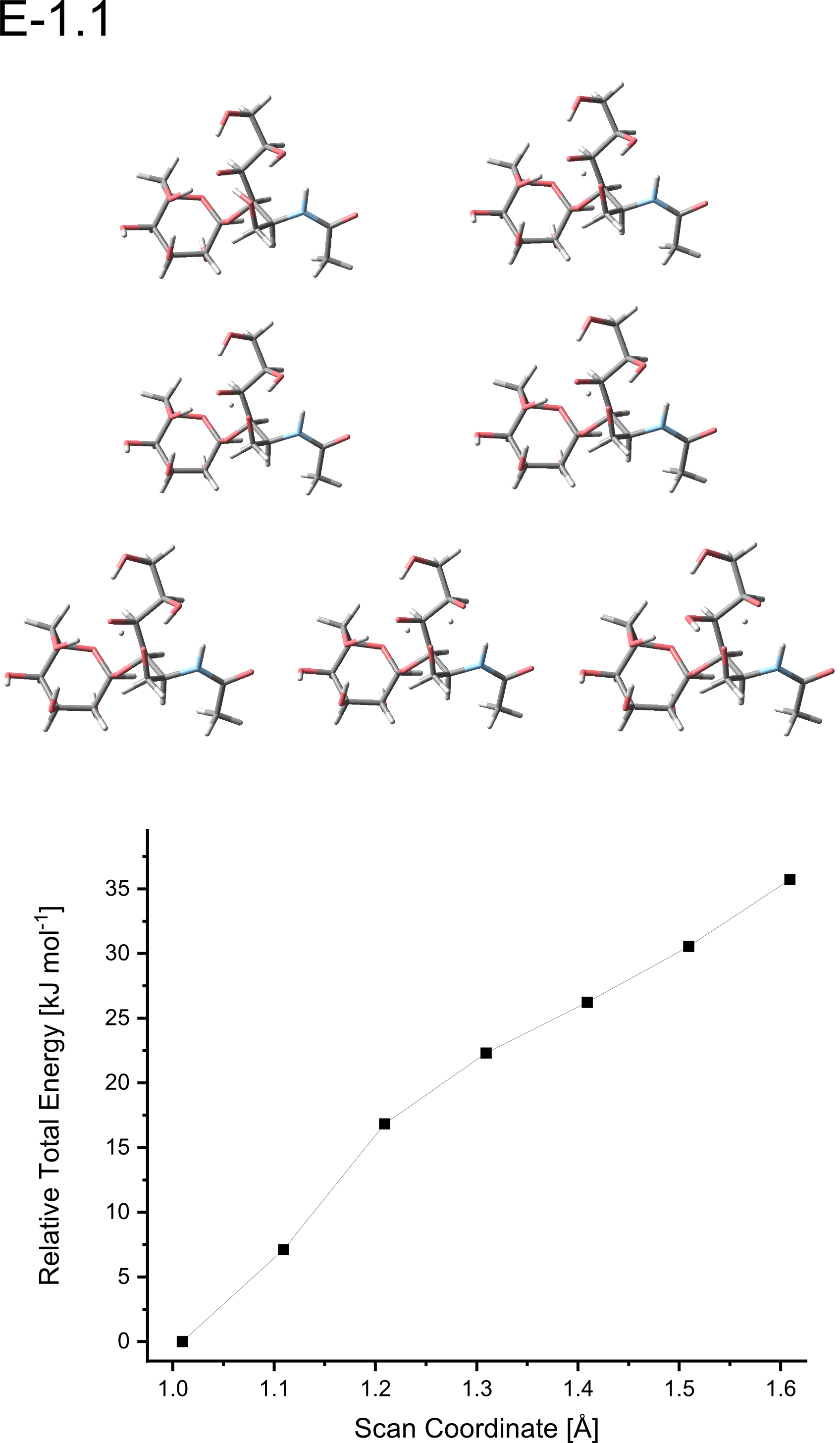
**

Figure S13. Relaxed potential energy surface scans for the first internal proton transfer process in conformer E-1. Total energies are relative to the initial structure.


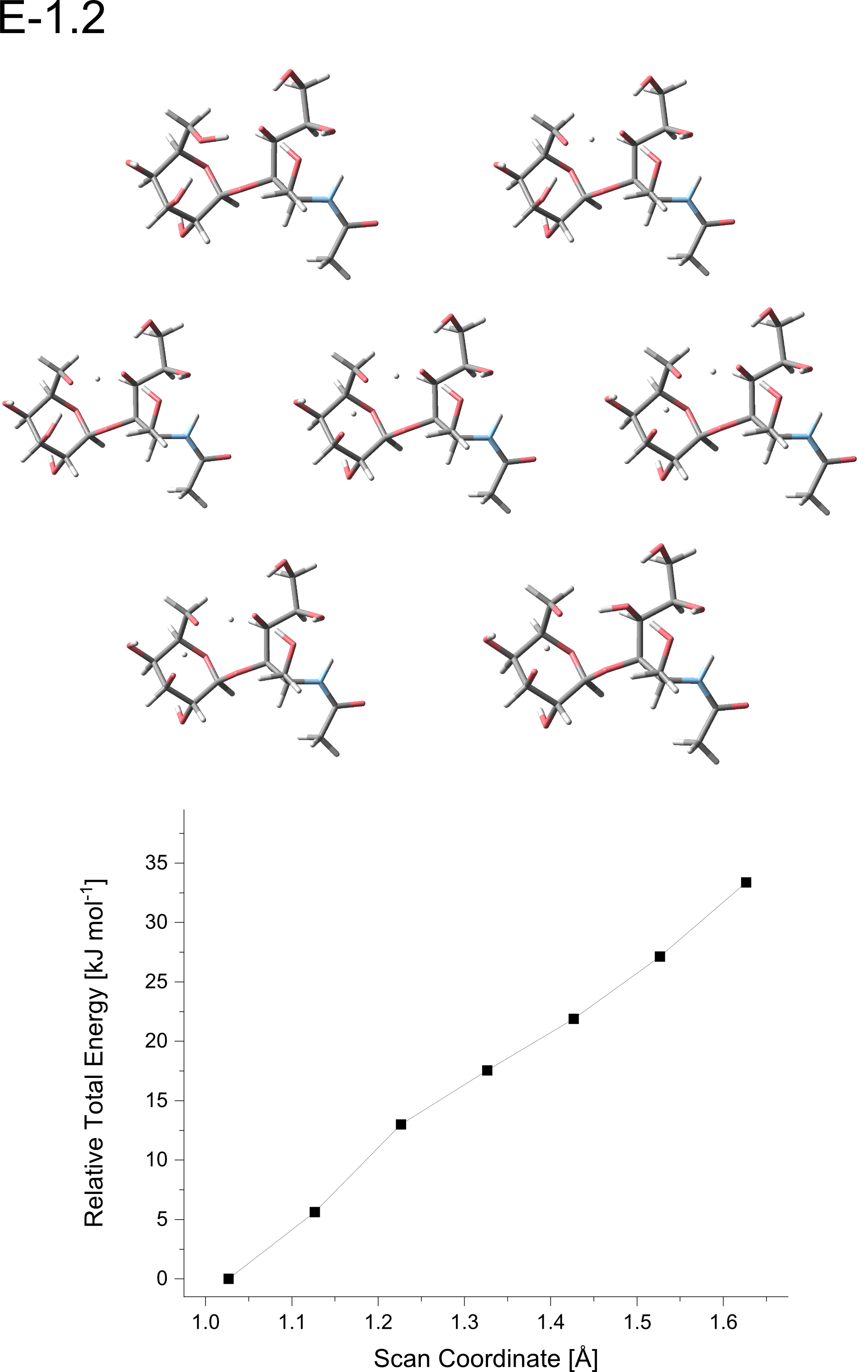


Figure S14. Relaxed potential energy surface scans for the second internal proton transfer process in conformer E-1. Total energies are relative to the initial structure.


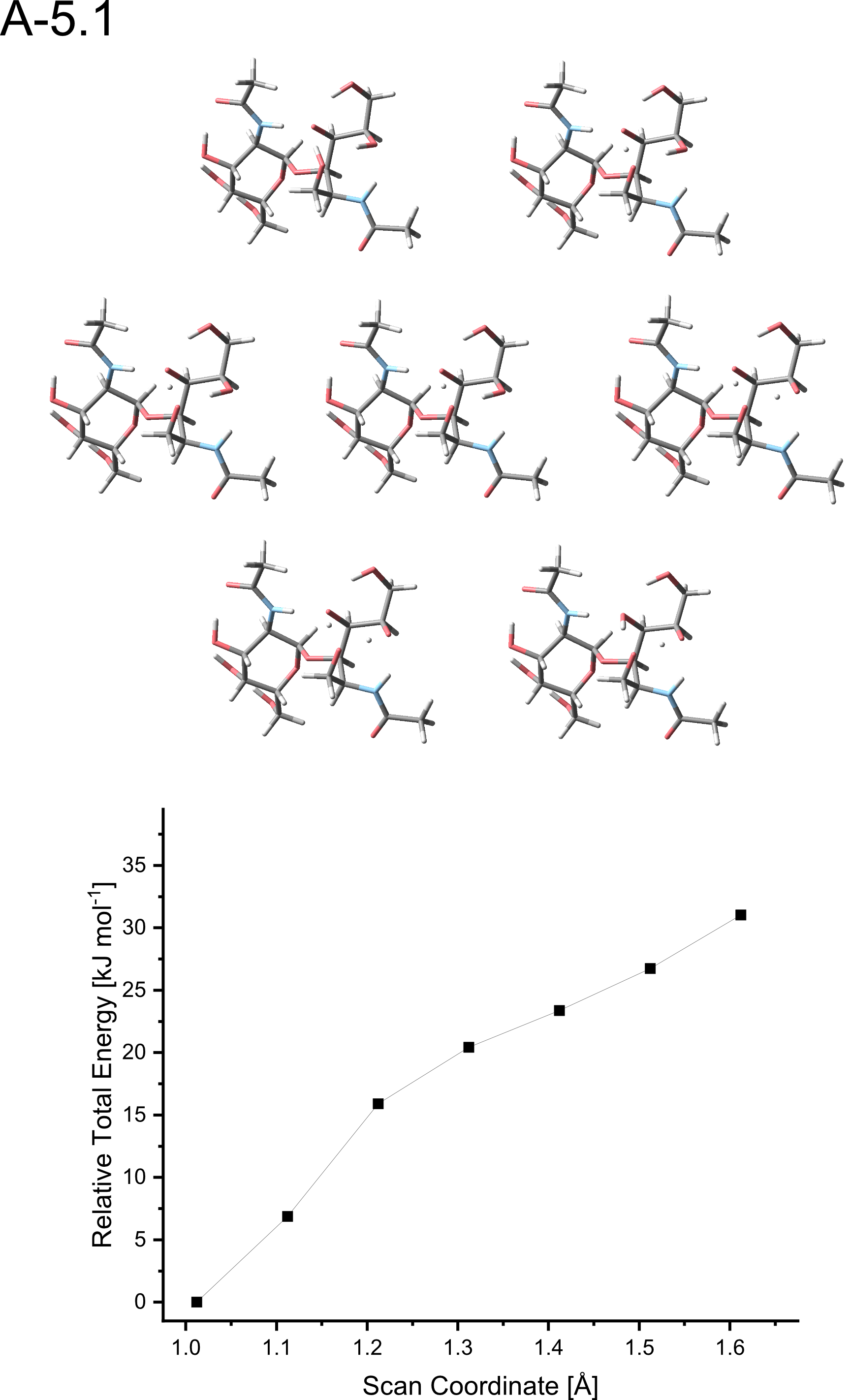


Figure S15. Relaxed potential energy surface scans for the first internal proton transfer process in conformer A-5. Total energies are relative to the initial structure.


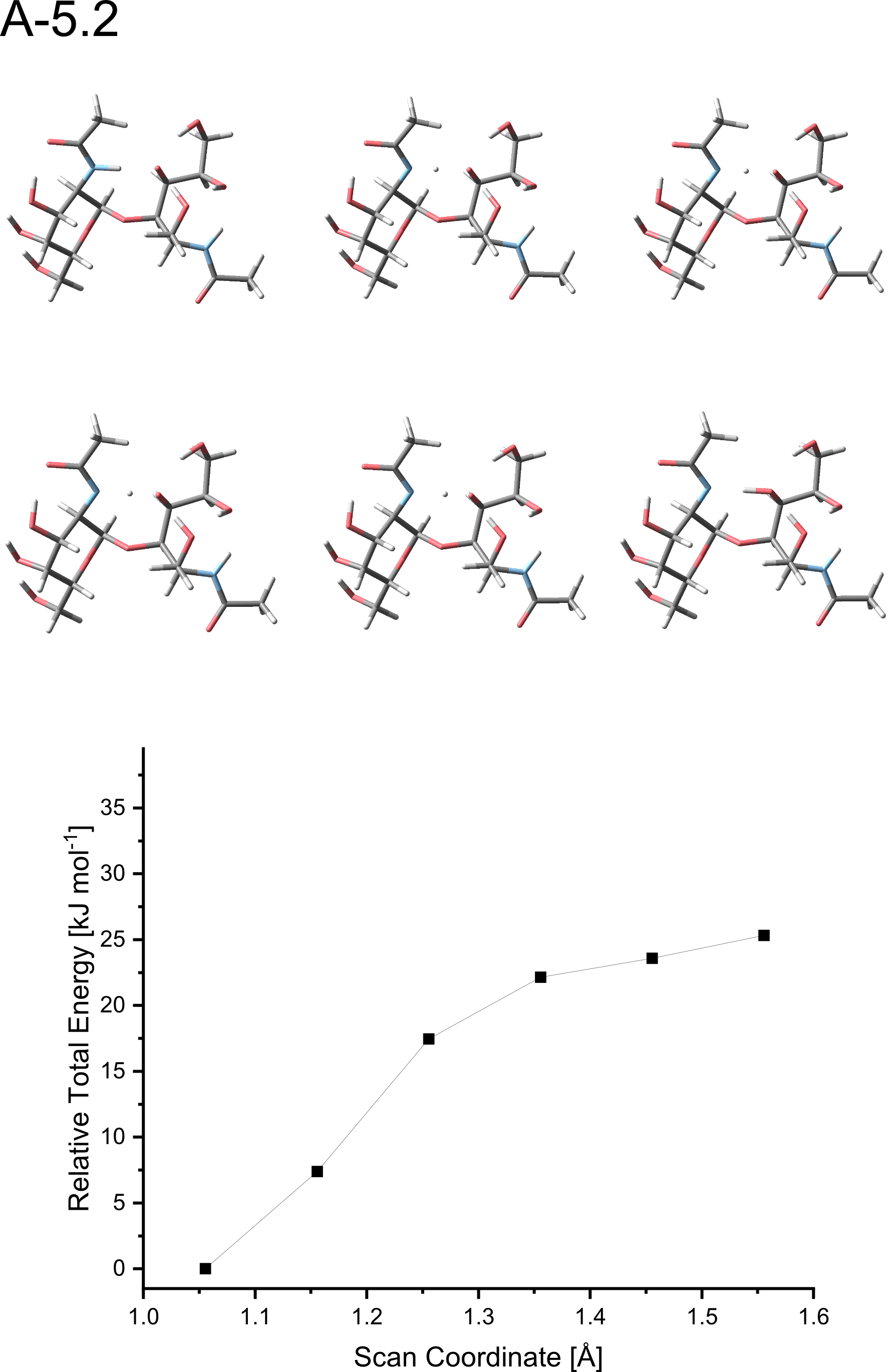


Figure S16. Relaxed potential energy surface scans for the second internal proton transfer process in conformer A-5. Total energies are relative to the initial structure.


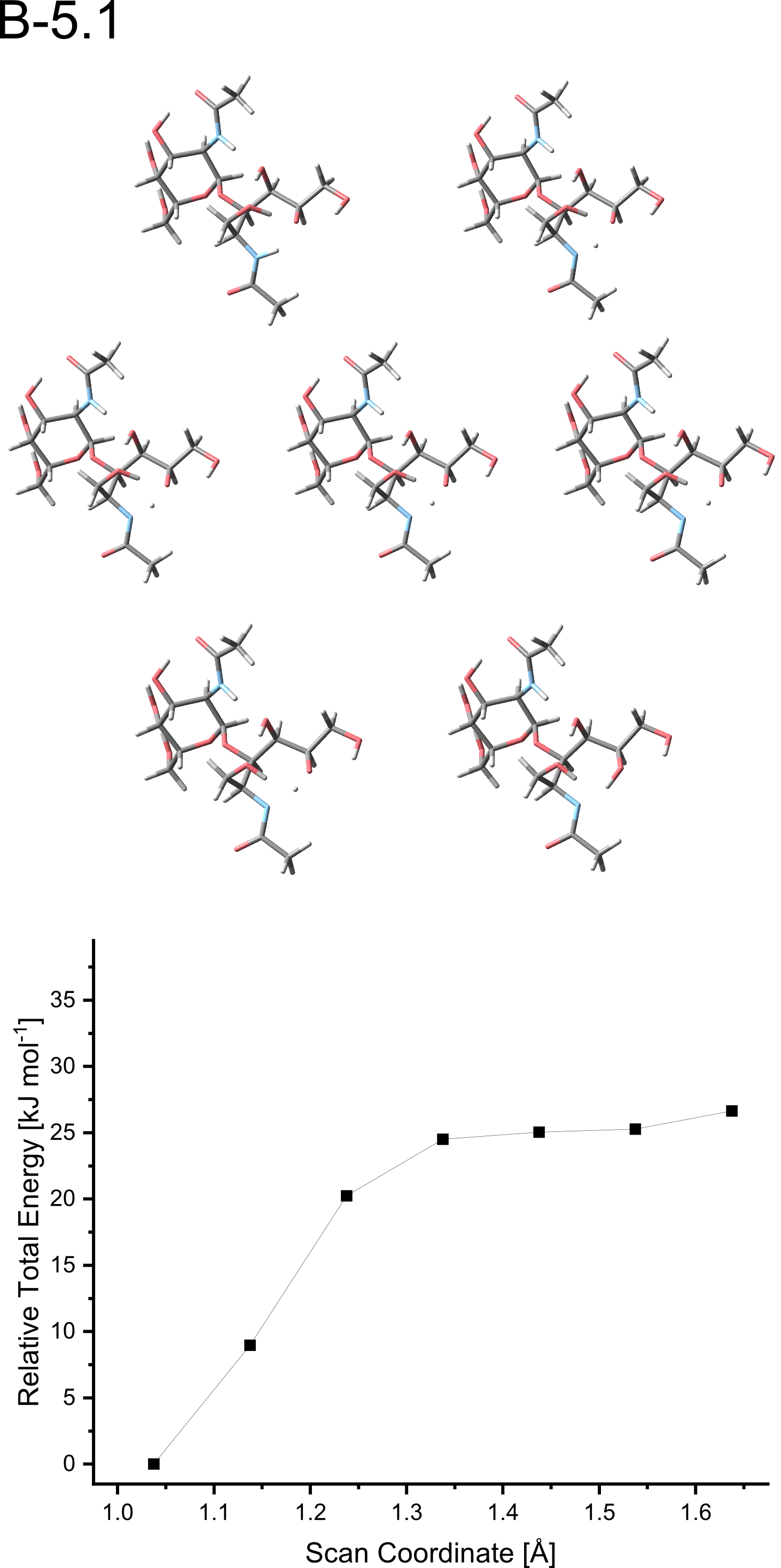


Figure S17. Relaxed potential energy surface scans for the first internal proton transfer process in conformer B-5. Total energies are relative to the initial structure.


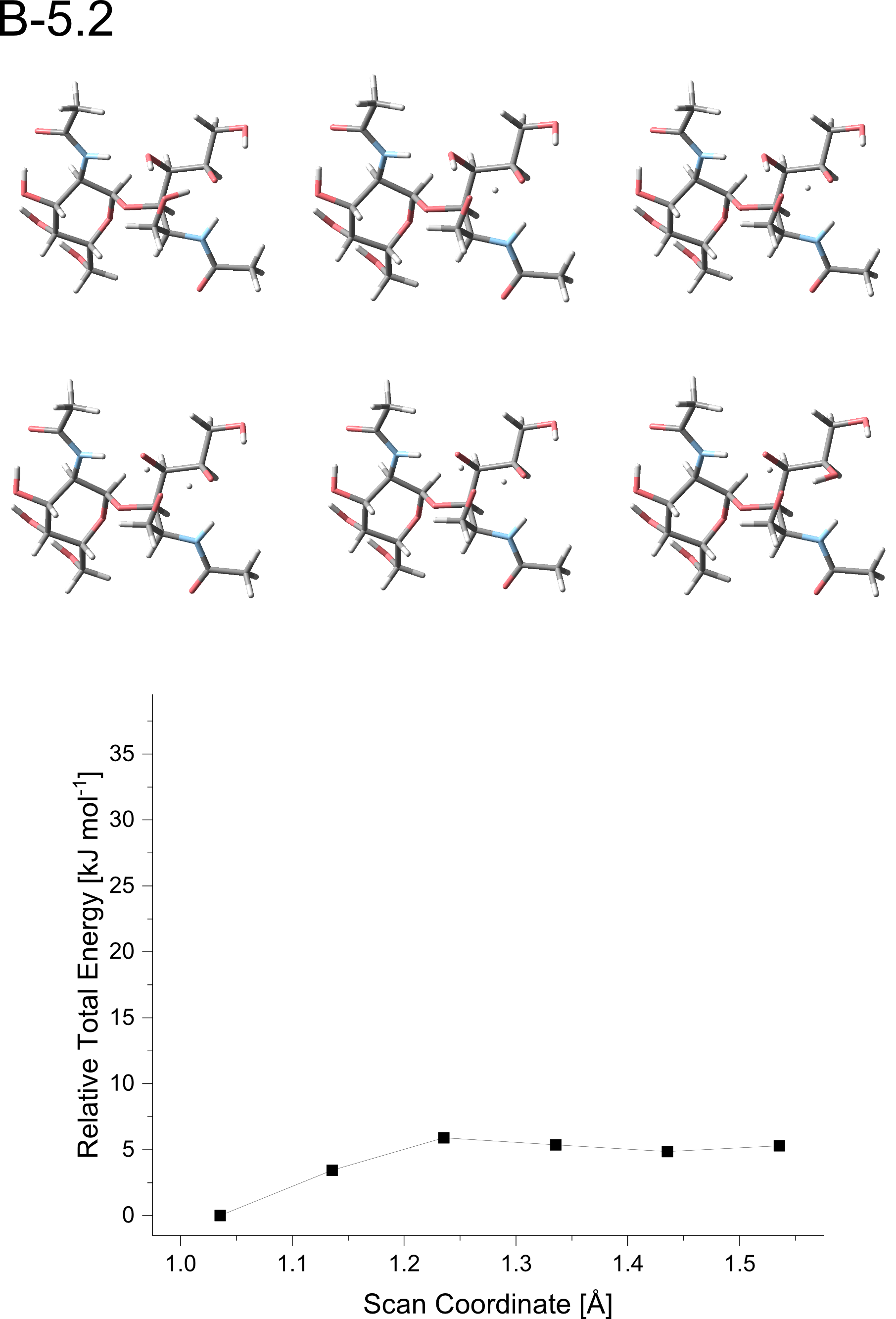


Figure S18. Relaxed potential energy surface scans for the second internal proton transfer process in conformer B-5. Total energies are relative to the initial structure.


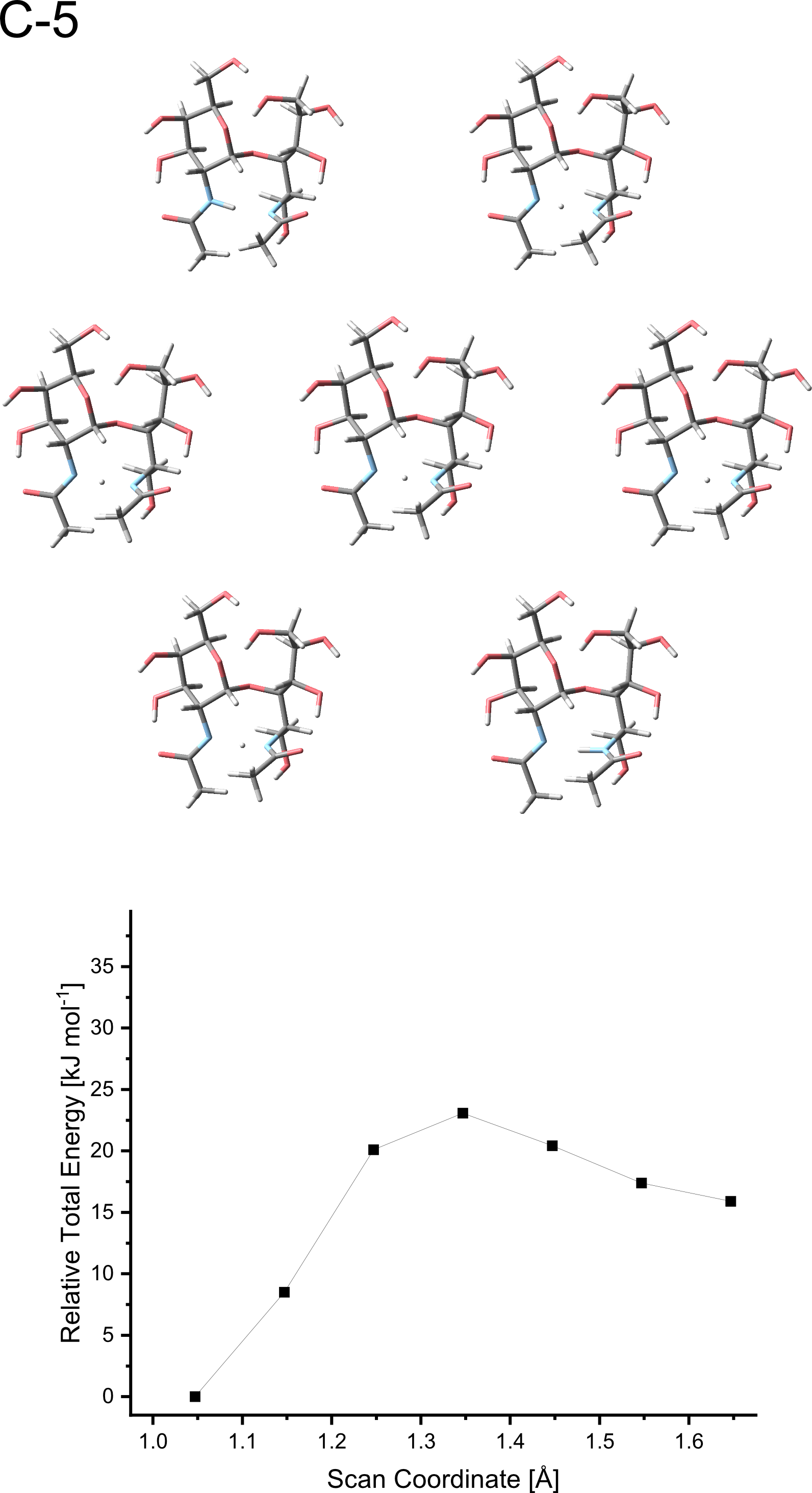


Figure S19. Relaxed potential energy surface scans for the internal proton transfer process in conformer C-5. Total energies are relative to the initial structure.


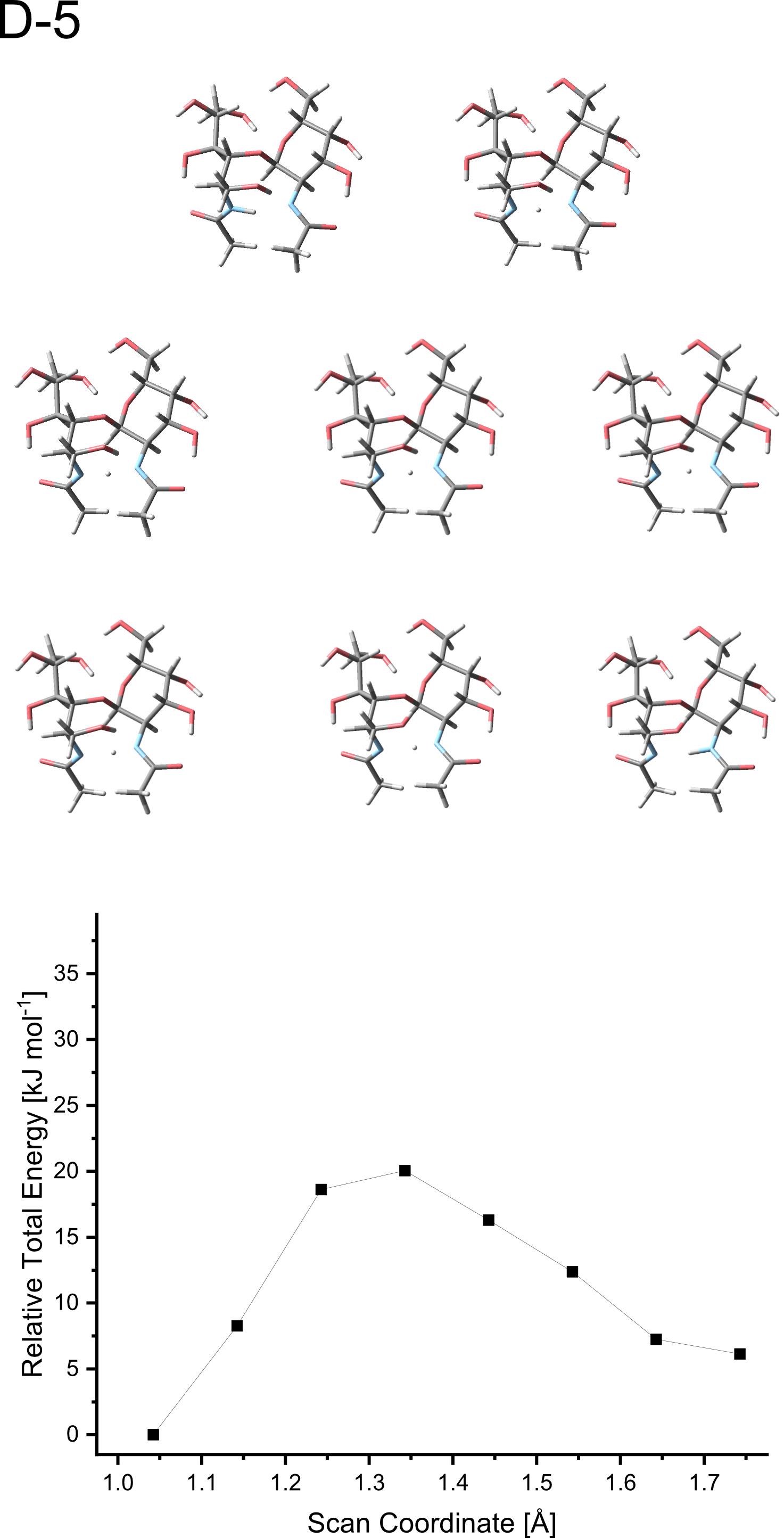


Figure S20. Relaxed potential energy surface scans for the internal proton transfer process in conformer D-5. Total energies are relative to the initial structure.


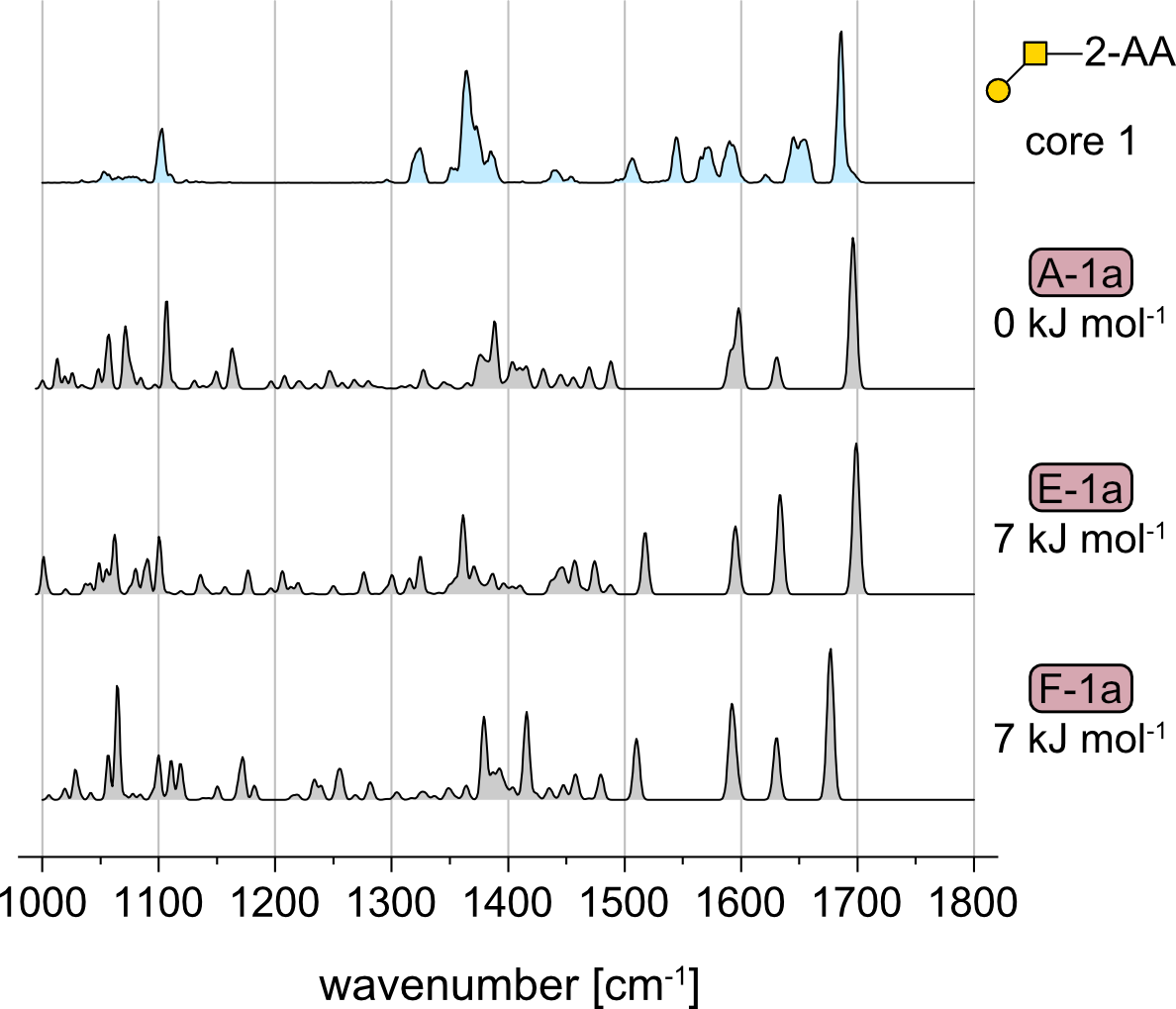


Figure S21. Comparison of experimental (blue trace) and theoretical spectra (gray traces) for deprotonated core 1 labeled with 2-aminobenzoic acid (2-AA). All conformers are deprotonated at carboxylic acid group of the 2-AA label. Relative free energies at 90 K of the individual deprotomers are indicated.


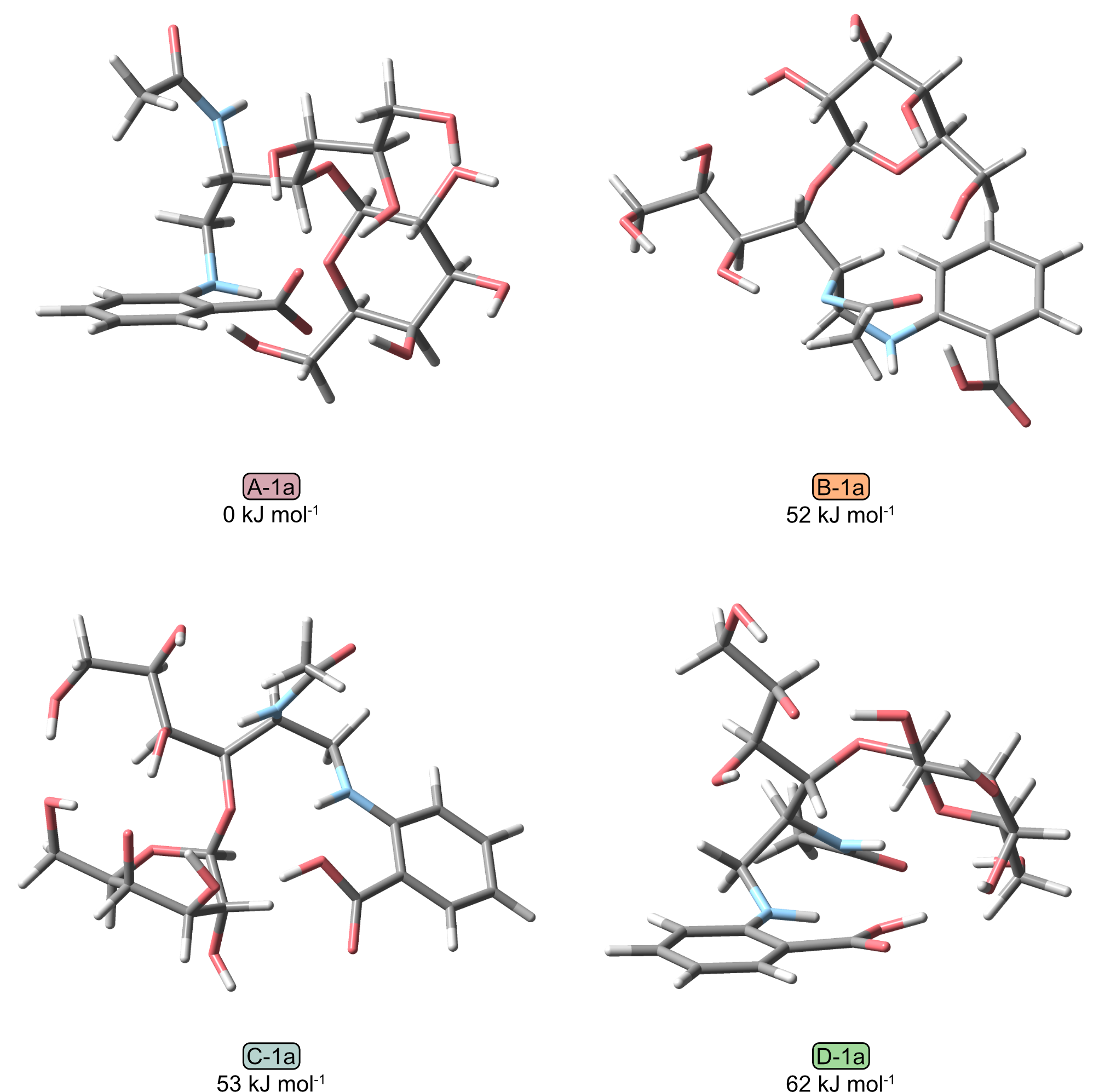


Figure S22. Computed structures of low-energy deprotomers for deprotonated core 1 labeled with 2-AA. Relative free energies at 90 K are indicated.


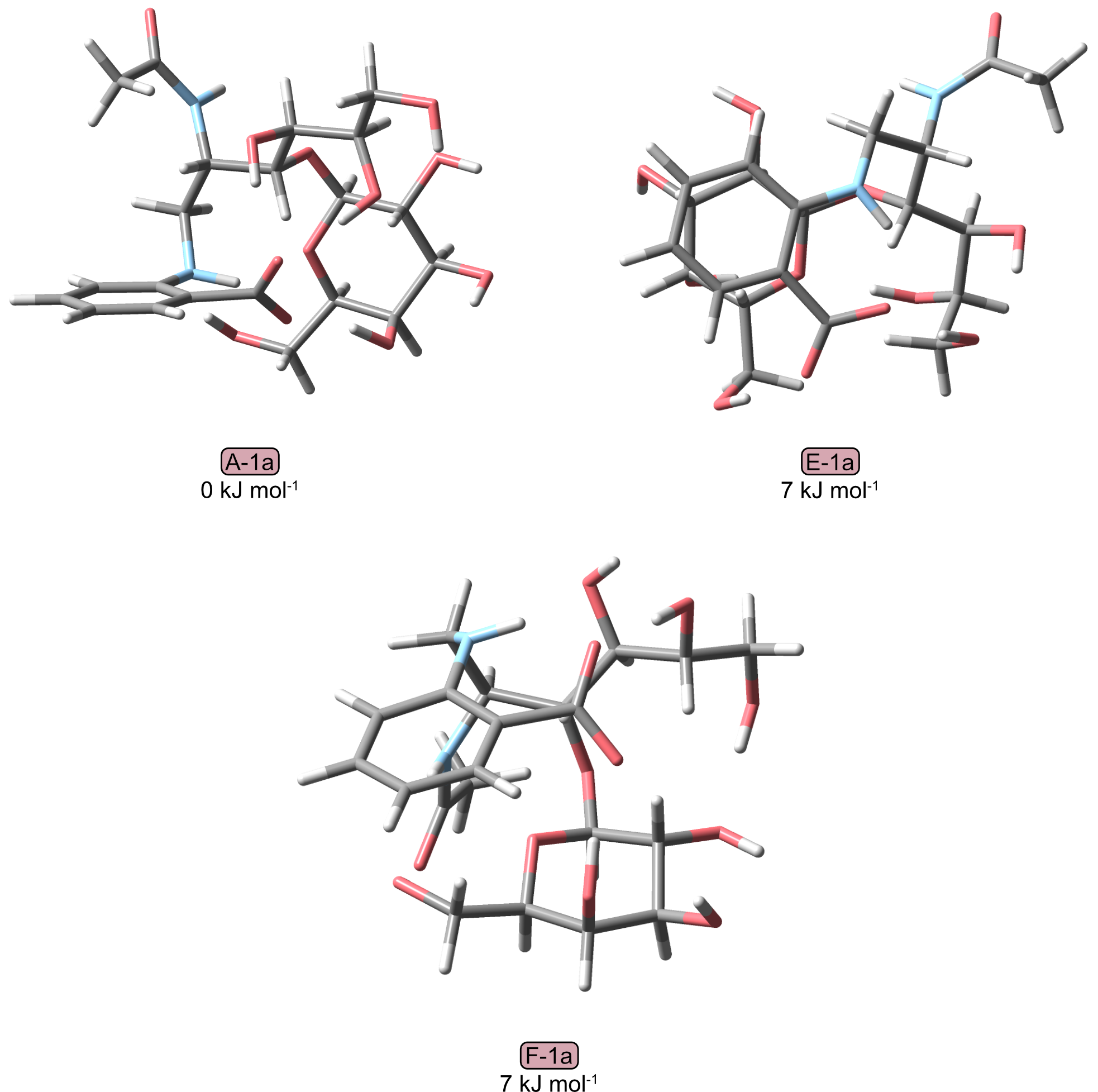


Figure S23. Computed structures of low-energy conformers for core 1 labeled with 2-AA deprotonated at the carboxylic acid group. Relative free energies at 90 K are indicated.

**xyz-Coordinates of Reoptimized Structures**

xyz-Coordinates of all reoptimized geometries at the CAM-B3LYP+D3BJ/6-311+G(d,p) level of theory can be found in a separate document “coordinates.xyz”.

**References**

[1] A. I. González Flórez, E. Mucha, D.-S. Ahn, S. Gewinner, W. Schöllkopf, K. Pagel, G. von Helden, *Angew. Chem. Int. Ed.* **2016**, *55*, 3295-3299.

[2] E. Mucha, A. I. González Flórez, M. Marianski, D. A. Thomas, W. Hoffmann, W. B. Struwe, H. S. Hahm, S. Gewinner, W. Schöllkopf, P. H. Seeberger, G. von Helden, K. Pagel, *Angew. Chem. Int. Ed.* **2017**, *56*, 11248-11251.

[3] U. Even, *EPJ Tech. Instrum.* **2015**, *2*, 17.

[4] W. Schöllkopf, S. Gewinner, H. Junkes, A. Paarmann, G. von Helden, H. Bluem, A. M. Todd, *Proc. SPIE* **2015**, *9512*, 95121L.

[5] M. Götze, L. Polewski, L. Bechtella, K. Pagel, *J. Am. Soc. Mass Spectrom.* **2023**, *34*, 2403-2406.

[6] L. Bechtella, J. Chunsheng, K. Fentker, G. R. Ertürk, M. Safferthal, Ł. Polewski, M. Götze, S. Y. Graeber, G. M. Vos, W. B. Struwe, M. A. Mall, P. Mertins, N. G. Karlsson, K. Pagel, *Nat. Commun.* **2024**, *15*, 2611.

[7] K. Pagel, D. J. Harvey, *Anal. Chem.* **2013**, *85*, 5138-5145.

[8] J. Hofmann, W. B. Struwe, C. A. Scarff, J. H. Scrivens, D. J. Harvey, K. Pagel, *Anal. Chem.* **2014**, *86*, 10789-10795.

[9] P. Pracht, F. Bohle, S. Grimme, *Phys. Chem. Chem. Phys.* **2020**, *22*, 7169-7192.

[10] C. Bannwarth, S. Ehlert, S. Grimme, *J. Chem. Theory Comput.* **2019**, *15*, 1652-1671.

[11] R. Krishnan, J. S. Binkley, R. Seeger, J. A. Pople, *J. Chem. Phys.* **1980**, *72*, 650-654.

[12] T. Yanai, D. P. Tew, N. C. Handy, *Chem. Phys. Lett.* **2004**, *393*, 51-57.

[13] S. Grimme, S. Ehrlich, L. Goerigk, *J. Comp. Chem.* **2011**, *32*, 1456-1465.

[14] G. W. T. M. J.Frisch, H. B. Schlegel, G. E. Scuseria, M. A. Robb, J. R. Cheeseman, G. Scalmani, V. Barone, G. A. Petersson, H. Nakatsuji, X. Li, M. Caricato, A. V. Marenich, J. Bloino, B. G. Janesko, R. Gomperts, B. Mennucci, H. P. Hratchian, J. V. Ortiz, A. F. Izmaylov, J. L. Sonnenberg, D. Williams-Young, F. Ding, F. Lipparini, F. Egidi, J. Goings, B. Peng, A. Petrone, T. Henderson, D. Ranasinghe, V. G. Zakrzewski, J. Gao, N. Rega, G. Zheng, W. Liang, M. Hada, M. Ehara, K. Toyota, R. Fukuda, J. Hasegawa, M. Ishida, T. Nakajima, Y. Honda, O. Kitao, H. Nakai, T. Vreven, K. Throssell, J. A. Montgomery Jr., J. E. Peralta, F. Ogliaro, M. J. Bearpark, J. J. Heyd, E. N. Brothers, K. N. Kudin, V. N. Staroverov, T. A. Keith, R. Kobayashi, J. Normand, K. Raghavachari, A. P. Rendell, J. C. Burant, S. S. Iyengar, J. Tomasi, M. Cossi, J. M. Millam, M. Klene, C. Adamo, R. Cammi, J. W. Ochterski, R. L. Martin, K. Morokuma, O. Farkas, J. B. Foresman and D. J. Fox, Revision A.03 ed., Gaussian Inc., Wallingford CT, **2016**.

[15] U. C. Singh, P. A. Kollman, *J. Comp. Chem.* **1984**, *5*, 129-145.

[16] M. F. Mesleh, J. M. Hunter, A. A. Shvartsburg, G. C. Schatz, M. F. Jarrold, *J. Phys. Chem.* **1996**, *100*, 16082-16086.

[17] L. Zanotto, G. Heerdt, P. C. T. Souza, G. Araujo, M. S. Skaf, *J. Comput. Chem.* **2018**, *39*, 1675-1681.

[18] T. Lu, F. Chen, *J. Comput. Chem.* **2012**, *33*, 580-592.

[19] T. Lu, *J. Chem. Phys.* **2024**, *161*, 082503.

[20] W. Humphrey, A. Dalke, K. Schulten, *J. Mol. Graph.* **1996**, *14*, 33-38.
